# Layered Tin Chalcogenides SnS and SnSe: Lattice Thermal Conductivity Benchmarks and Thermoelectric Figure of Merit

**DOI:** 10.1021/acs.jpcc.2c02401

**Published:** 2022-08-16

**Authors:** Jordan Rundle, Stefano Leoni

**Affiliations:** Materials Discovery Group, School of Chemistry, Cardiff University, C10 3AT Cardiff, U.K.

## Abstract

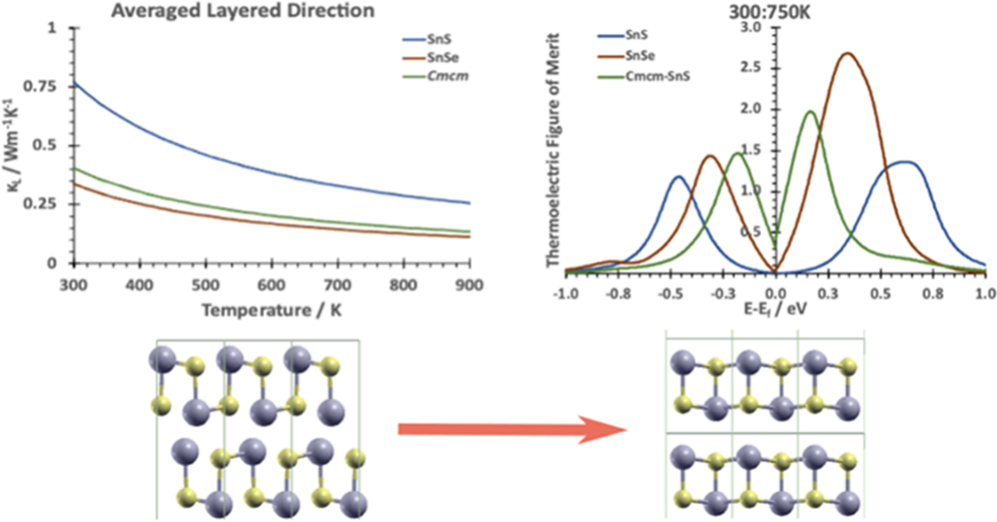

Tin sulfide (SnS) and tin selenide (SnSe) are attractive materials for thermoelectric conversion applications. Favorable small band gap, high carrier mobility, large Seebeck coefficient, and remarkably low lattice thermal conductivity are a consequence of their anisotropic crystal structure of symmetry **Pnma**, made of corrugated, black phosphorus-like layers. Their internal lattice dynamics combined with chemical bond softening in going from SnS to SnSe make for subtle effects on lattice thermal conductivity. Reliable prediction of phonon transport for these materials must therefore include many-body effects. Using first principles methods and a transferable tight-binding potential for frozen phonon calculations, here, we investigate the evolution of thermal lattice conductivity and thermoelectric figure of merit in **Pnma**-SnS and -SnSe, also including the high-temperature **Cmcm**-SnS phase. We show how thermal conductivity lowering in SnS at higher temperatures is largely due to dynamic phonon softening ahead of the **Pnma**–**Cmcm** structural phase transition. SnS becomes more similar to SnSe in its lifetime and mean free path profiles as it approaches its high-temperature **Cmcm** phase. The latter nonetheless intrinsically constraints phonon group velocity modules, preventing SnS to overtake SnSe. Our analysis provides important insights and computational benchmarks for optimization of thermoelectric materials via a more efficient computational strategy compared to previous ab initio attempts, one that can be easily transferred to larger systems for further thermoelectric materials nanoengineering. The good description of anharmonicity at higher temperatures inherent to the tight-binding potential yields calculated lattice conductivity values that are in very good agreement with experiments.

## Introduction

The increased energy demand and migration away from fossil fuel combustion call for innovative material solutions for sustainable development. Automotive exhaust and industrial processes waste about two-thirds of the total energy into heat. Furthermore, harvesting heat from battery packs, fuel, and photovoltaic cells increases the overall device energy conversion efficiency and process sustainability and circularity. Finding high-performance thermoelectric materials capable of directly and reversibly converting heat to electrical energy is therefore a task of top priority.^1−7^ The efficient control of thermoelectric energy conversion processes entails the ability to assemble materials with tailored thermal transport properties.^8^ The thermoelectric efficiency at a given temperature is expressed by the dimensionless figure of merit *ZT* = *S*^2^σ*T*/κ. Here, *S* is the Seebeck coefficient, σ is the electrical conductivity, and κ is the thermal conductivity, which is the sum of lattice and electronic contributions κ = κ_latt_ + κ_e_. This expression suggests a large Seebeck coefficient and high electrical conductivity as requirements for good thermoelectric materials, as well as low thermal conductivity. Nonetheless, these conditions cannot be immediately translated into a material design strategy due to the interdependence of the physical parameters contributing to *ZT*.

With lead chalcogenides firmly established in the literature as strong thermoelectrics,^6,9,10^ tin chalcogenides have emerged as suitable replacements exhibiting lower toxicity than Pb-based materials. Binary tin chalcogenides SnX (X = S, Se) owe their versatility and property tunability to their distorted NaCl geometry, which yields an anisotropic layered structure of the **Pnma** symmetry. With a small band gap, high carrier mobility, a large Seebeck coefficient, and extremely low lattice thermal conductivity, SnSe has been the focus of an intense search for high-figure-of-merit materials.^11^ With values as low as 0.25 W m^–1^ K^–1^ above 800 K, SnSe outperforms top composite thermoelectric materials, without any need for grain engineering,^12^ nanoinclusions, or nanostructuring. Morphology, vacancies, and synthetic approaches can sensibly affect thermal conductivity.^13,14^ At high temperatures, both **Pnma**-SnS and -SnSe undergo a structural phase transition to a **Cmcm** structure, in which the *c-*axis shortens to more closely resemble the *b*-axis. This follows from the compression of the intrinsically deformable, zig-zag “accordion-like” layers’ geometry and from antiparallel layer displacements. Interestingly, it is for temperatures near to and above the phase transition threshold that the highest *ZT* figures are measured. While low lattice thermal conductivities can be expected from the anisotropic, layered structure alone,^15^ the relevance of several structures toward enhancing thermoelectricity is therefore very intriguing. On crossing the phase boundary, favorable electronic properties are maintained, while layer compression and reshuffling further assist phonon softening.^15^ Detailed diffraction studies suggest simultaneous positive and negative Seebeck effects along different directions as a consequence of structural changes induced by pressure.^16^ Low thermal transport in **Cmcm**-SnS is associated with phonon softening and enhanced three-phonon scattering, affecting in particular low-frequency modes.^17^

The lighter homologue SnS has a comparatively smaller thermoelectric figure of merit at lower temperatures, while ab initio calculations suggest the **Cmcm** phase to be competitive in SnSe at higher temperatures,^18^ confirming the subtle structure–property relationship at work in this class of materials.^19^ Pressure strongly affects transport in SnS, inducing superconductivity, an effect already noticed in black phosphorus and associated with an electronic fingerprint of a good thermoelectric response.^20,21^

Heat transport properties of SnS and SnSe, individually or comparatively, have been calculated from first principles in several contexts.^15,17−19,22−27^ The interplay of the anisotropic structure, temperature-induced phonon softening, and many-body phonon scattering has been indicated as an important factor in explaining remarkably low lattice conductivities.^15,17,25^ While both **Pnma**-SnS and -SnSe are consistently evaluated as poor thermal conductors, their relative ranking has been more challenging to determine from first principles calculations.^23^

In the search for top-performing thermoelectrics, complex materials are indicated as technology relevant, comprising nanoincluded composites, mesoscale grain boundaries, or superlattice engineering to effectively act as phonon scatterers.^8,10^ To further improve on the figure of merit of tin chalcogenides, it is therefore desirable to have a reliable yet computationally efficient baseline for thermal transport benchmark calculations, one that can be easily implemented on a larger structural scale, for which ab initio approaches are impractical. As with all thermoelectric materials, the question of doping is also pertinent, and obtaining sufficient doping levels without detriment to the lattice dynamics is a delicate task. Dopants commonly used are sodium^28^ and cadmium^13^ for p-type and chlorine^29^ for n-type.

In this work, we set such a baseline by electronic and thermal transport in **Pnma-**SnS and -SnSe, including a comparison with **Cmcm**-SnS. The electronic part of the thermoelectric figure of merit is obtained from the Boltzmann transport equation based on the Wannier function (WF) interpolation of the electronic bands.^30^ Phonon frequencies are calculated from a transferable tight-binding interatomic potential,^31^ while lattice thermal conductivity is obtained from the linearized phonon Boltzmann equation (LBTE) using a supercell approach.^32,33^ Thermal transport anisotropy and ranking are obtained in good agreement with experiments, while the increasing similarity of SnS and SnSe is mapped onto a common **Cmcm** structural motif, which becomes dynamically important at high temperatures.

## Methods

### Electronic Structure and Phonons

Bloch states were calculated using density functional theory in the GGA-PBE approximation, using Vanderbilt ultrasoft Sn, S, and Se standard solid-state pseudopotentials (SSSP,^34^ efficiency branch) available from the materials cloud (materialscloud.org). Pseudopotentials included 5s^2^ 5p^2^ 4d^10^ for Sn and 3s^2^ 3p^2^ for S and Se. The plane-wave expansion of valence electron wave functions and charge density used kinetic energy cutoffs of 70 and 560 Ry, respectively. Calculations were performed using the pw.x code of the Quantum Espresso (QE) package v. 6.7.^35^ SCF calculations were run on a k-grid of 3 × 8 × 8 data points. Cell parameters and atomic coordinates were relaxed below 1 × 10^–8^ Ry and 1 × 10^–6^ Ry/Bohr in energy and forces, respectively. Bloch states and overlaps for maximally localized WF (MLWF) calculations were evaluated on a Monkhorst–Pack 4 × 11 × 10 **k**-point mesh for both SnS and SnSe.

A tight-binding band structure model of Bloch functions was obtained from interpolating MLWFs, calculated with the Wannier90 package.^36^ As an initial guess for Wannier functions, p orbitals were used for Sn, S, and Se (24 MLWFs), and s projectors were included for Sn, excluding energetically low lying 3s bands (28 MLWF model). Iterative spread minimization provided real-valued, maximally localized WFs using a convergence tolerance of 1.0 × 10^–10^ Ω.

The Seebeck tensor ***S*** depends on the doping level of the material through the chemical potential μ and the temperature *T*. Collisions in the semiclassical Boltzmann transport equation are mapped into band *n* and **k** vector-specific relaxation times, τ_n**k**_. In the single relaxation approximation, a constant relaxation time is assumed for every band and momentum, τ = τ_n**k**_.

The postw90 package of Wannier90 was used to interpolate band velocities *v* and calculate the transport distribution function  (TDF)^30^ on a finer **k**-mesh of 250 points per reciprocal Angstrom, 22 × 62 × 56 for SnS and 22 × 60 × 54 for SnSe. μ values were chosen in a range of −3 to +3 eV from the Fermi energy, in steps of 0.001 eV.

Electrical conductivity **σ**, Seebeck coefficient **S**, and electronic thermal conductivity **κ** = **K** – **σS**^**2**^T tensors were calculated semiclassically with the Boltzmann transport equation, using the BoltzWann module of Wannier90.^30^ The dependency on temperature and chemical potential is given as follows:
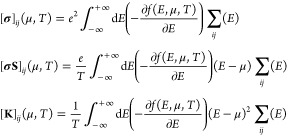
The calibration of the relaxation time was made on the initial transport calculation with unitary τ = 1 fs. For SnS, a chemical potential μ of −0.31635 eV matched the experimental range of 704 μV K^–1^ along the *b-*axis and 709 μV K^–1^ along the *c-*axis^37^ (calculated values were 682 and 729 μV K^–1^, respectively). μ values were chosen by minimizing the squared deviation |*S*(μ)_a_^calcd^ – *S*_a_^exp^|^2^ + |*S*(μ)_b_^calcd^ – *S*_b_^exp^|^2^ of the calculated and experimental Seebeck coefficients along different axes. The relaxation time was then adjusted at this μ value to closely reproduce conductivity values of 2.26 and 1.95 S cm^–1^ along the *b-* and *c-*axis, respectively^37^ (calculated values of 2.76 and 1.62 S cm^–1^, respectively), yielding τ = 24.9394 fs. For SnSe, Seebeck coefficients of 533 μV K^–1^ along the *b*-axis and 482 μV K^–1^ along the *c*-axis (505 and 512 μV K^–1^ from ref (38), respectively) imply that μ = −0.21905 eV. With τ = 20.1182 fs, the corresponding electronic conductivities are 9.98 and 13.20 S cm^–1^, respectively (10.52 S cm^–1^ along the *b*-axis and 12.47 S cm^–1^ along the *c*-axis^38^).

### Thermal Conductivity

The linearized phonon Boltzmann transport equation (LBTE) was solved using the single-mode relaxation approximation (SMRT/RTA).^32,33^ Therein, the lattice thermal conductivity contributed by phonon modes was calculated from mode heat capacity, phonon group velocity, and relaxation time according to 

The mode heat capacity *C*_λ_ for phonon frequency ω_λ_ is given as follows
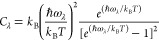
The many-body nature of the scattering process introduces three-phonon interaction contributions to phonon mode lifetimes. The corresponding three-phonon interaction strengths can be computed from phonon harmonic frequencies, displacement eigenvectors, and third-order force constant matrices.^32,33^ The imaginary part of the self-energy, computed from three-body phonon scattering, takes the form
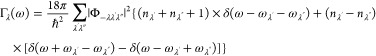
Φ_λλ′λ″_ is the interaction strength between three scattering λ, λ′, and λ″ phonons, while *n*_λ_ is the temperature-dependent equilibrium phonon occupation number, which is given as follows:
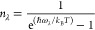
Γ_λ_ (ω) relates to the phonon lifetime as , where  is the phonon linewidth of the λ phonon mode.

For the phonon and lattice thermal conductivity calculations, PHONOPY^39^ and PHONO3PY^32^ software packages were employed.

In the SMRT/RTA approach, it is assumed that phonon relaxation can be replaced by phonon lifetime. This approximation can be checked by performing a computational demanding but more accurate full solution of the LBTE, according to ref (40). This was done for both SnS and SnSe (**Pnma**) to assess the numerical quality of the SMRT/RTA model for this class of compounds (see Tables S1 and S2 in the Supporting Information, SI).

Second- and third-order force constants were calculated based on the supercell approach with finite atomic displacements of 0.03 Å.^32^ The use of larger supercells is in general important to compute phonon–phonon scattering channels with better accuracy, as spurious imaginary modes may appear at low frequencies around Γ. For the **Pnma** structures, we used a 3 × 3 × 3 supercell for third-order force calculations and a 4 × 8 × 8 supercell for second-order force constants. **Cmcm**-SnS used a 3 × 3 × 3 supercell for third-order force calculations and a 6 × 6 × 6 supercell for the second-order force constants. Quantum Espresso phonon calculations (PHONOPY) were based on a 2 × 4 × 4 supercell, while CP2K-xTB phonons used a 3 × 3 × 3 supercell. A large supercell number is required for the calculation of third-order force constants. While this task can be simplified by using a cutoff pair-distance, beyond which force contributions are vanishing, here we have used the full supercell range for all structures. Sampling meshes were produced using the automatic k-point generation inbuilt method. A value of 80 was used for the **Pnma** structures, equating to a k-mesh of 8 × 22 × 21 and 7 × 21 × 20 in **Pnma**-SnS and **Pnma**-SnSe, respectively. The Boltzmann transport equation was solved for several **q**-point grid densities until convergence (see Section S8 in the SI for details).

Supercell force constant calculations were based on the transferable tight-binding GFN-xTB^41^ scheme, as implemented in CP2K,^42^ using a modified interface to PHONO3PY. Geometry relaxed crystal structures were used to construct a supercell for the thermal conductivity calculation (see Figure S1 in the SI). Cell parameters and atomic positions were relaxed using the BFGS scheme on a k-grid of 6 × 8 × 8, including long-range contributions to the forces via Ewald summation.

## Results

### Crystal and Electronic Band Structures

**Figure 1:**
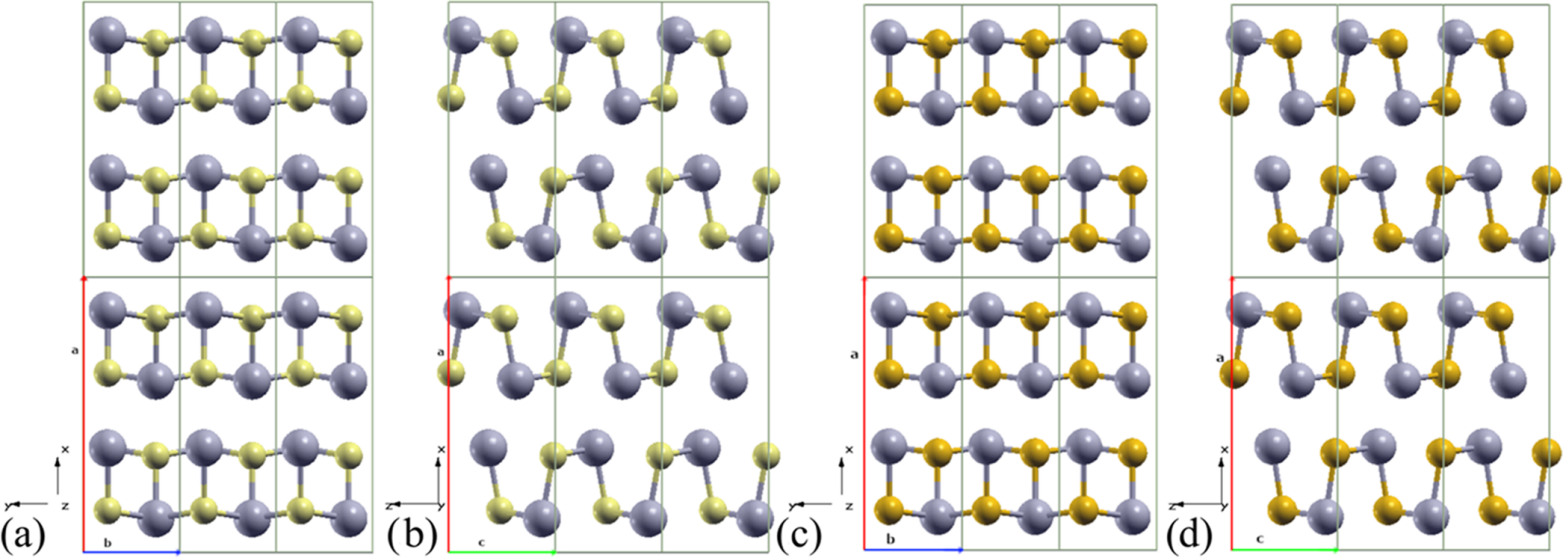
Relaxed crystal structures of *Pnma*-SnS and -SnSe, showing the open black phosphorus layer motif in the *ab* plane (SnS *a*, SnSe *c*) and *ac* plane (SnS *b*, SnSe *d*). The stacking direction in *Pnma* is along *a* and corresponds to the Cartesian *X* direction. The outer placement of Sn atoms within layers is visible. Sn is gray, S is yellow, and Se is orange. The axes of the standard orthorhombic unit cell are indicated (*a*, *b*, and *c* are red, blue, and green, respectively).

SnS and SnSe crystallize in the same GeS-type structure (B16, SG **Pnma**), which is a distorted NaCl (B1) structure, made of corrugated layers as in black phosphorus. The SnS lattice parameters after relaxation (QE, GGA-PBE) were *a* = 11.4455 Å, *b* = 4.0240 Å, and *c* = 4.4481 Å, consistent with experimental values of *a* = 11.200 Å, *b* = 3.987 Å, and *c* = 4.334 Å,^3^ as well as with previous calculations *a* = 11.433 Å, *b* = 4.024 Å, and *c* = 4.443 Å.^4^ Noticeable is the increased interlayer spacing by the very similar *b*-axis module (*b*/*a* calcd = 0.35, *b*/*a* exp = 0.36). The Sn–S polarized bond causes a sizable distortion of the layers compared to black phosphorus, pushing Sn to outer positions in the layers (see Figure 1). Similarly, SnSe relaxed into *a* = 11.7834 Å, *b* = 4.2054 Å, and *c* = 4.5638 Å, which are comparable to experiments (*a* = 11.501 Å, *b* = 4.153 Å, and *c* = 4.445 Å)^3^ and closely reflect previous plane-wave DFT-GGA results, *a* = 11.790 Å, *b* = 4.219 Å, and *c* = 4.524 Å.^7^ Again, the interlayer spacing is larger. For SnS, Sn–Sn distances represent the shortest interlayer contacts.

**Figure 2:**
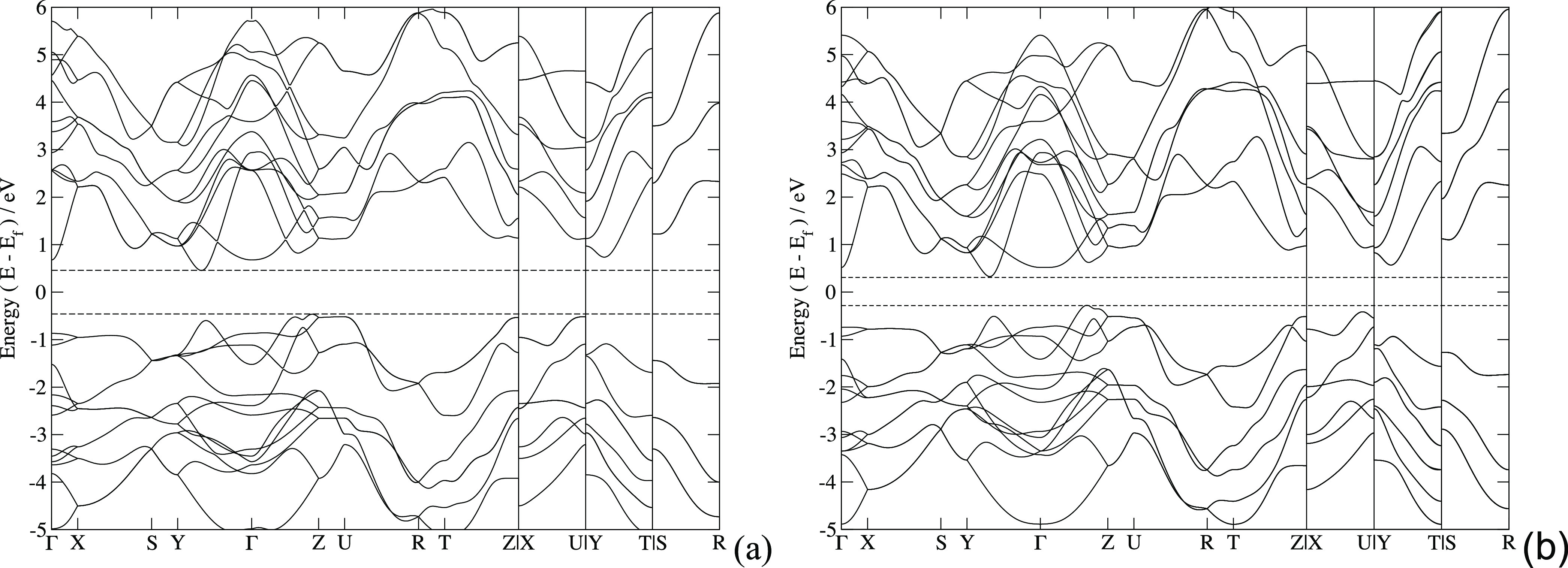
(a) SnS and (b) SnSe Wannier-interpolated band structures. The direct band gap for SnS is 1.08 eV, and the indirect band gap is 0.92 eV. For SnSe, the values are 0.86 and 0.61 eV, respectively.

The electronic band structures of SnS and SnSe are shown in Figure 2. The highest valence bands below the Fermi level show a characteristic “pudding mold”-like structure (Γ–*Z*) in SnS, which is also present in SnSe but with a more pronounced energy offset between the two maxima. A second valence band maximum is visible in correspondence with the conduction band minimum along *Y*–Γ. Their cusps connect two regions of linear *E*(**k**) dependence on **k**, like in an “opened Dirac cone.” The *Z*–U segment in SnS lies above the *Y*–Γ valence maximum cusp, which is not the case for SnSe, a feature also noticed in quasiparticle GW calculations.^25^ The second lowest CB minimum for both SnS and SnSe is found at Γ. Intralayer bands are more dispersive than interlayer ones. SnS shows a direct band gap of 1.08 eV and an indirect band gap of 0.92 eV, in agreement with literature values.^43,44^ For SnSe, the direct band gap is 0.86 eV and the indirect band gap is 0.61 eV (literature values are 0.98 and 0.582 eV, respectively;^43^ indirect band gap is 0.607 eV^45^).

### Phonons

Harmonic phonons enter the calculation of three-phonon interaction strengths. Previous ab initio phonon calculations^25^ indicate similar phonon band dispersions, with optical frequencies higher for SnS due to lighter S mass. The exceptionally low thermal conductivity of SnSe in particular is connected to optical modes softening.^11^ If on the one hand the crystal axis anisotropy of thermal transport is very well accounted for by ab initio approaches, comparison with experiments has been so far less conclusive regarding absolute values and relative ranking.^23^ To calculate phonons and thermal conductivity, we therefore based force calculations on a transferable, semiempirical third-order tight-binding potential, GFN-xTB^41^ as implemented in CP2K.^42^ The unit cell of SnS relaxed to *a* = 10.4111 Å, *b* = 3.7127 Å, and *c* = 3.8204 Å. Compared to experimental parameters, *a* = 11.200 Å, *b* = 3.987 Å, and *c* = 4.334 Å,^46^ the *a*-axis is more compressed, while *b*/*a* = 0.357 and *c*/*a* = 0.367; experimental values are *b*/*a* = 0.356 and *c*/*a* = 0.389. Corresponding geometries are given in Figure S1.

**Figure 3:**
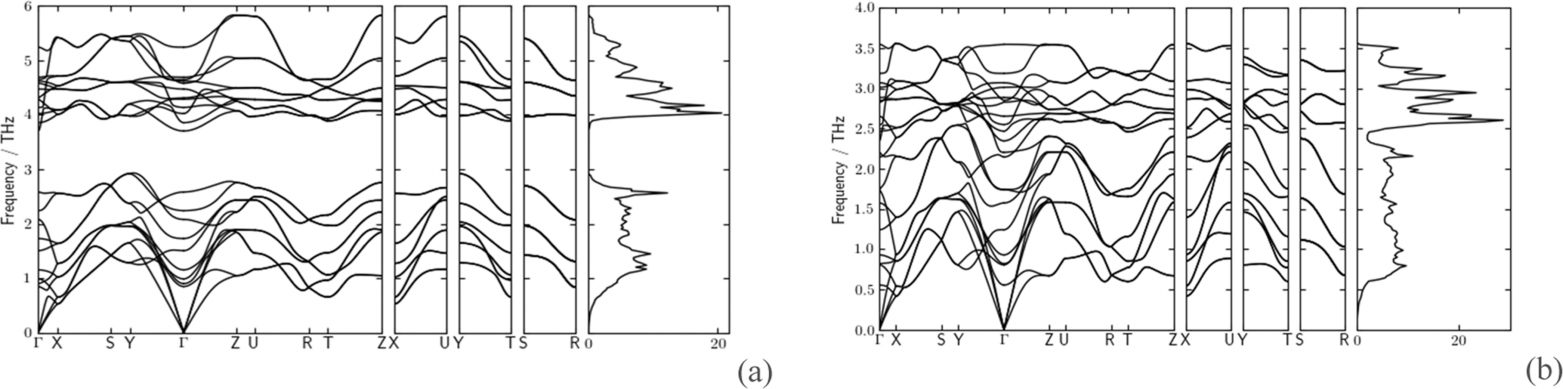
Phonon dispersion spectra and phonon density of states for (a) SnS and (b) SnSe, obtained with PHONOPY, using CP2K as a force driver. Third-order force constants are included. Linear acoustic phonons soften in the sequence Γ–*Y* (*b-*axis) > Γ–*Z* (*c-*axis) > Γ–*X* (*a-*axis).

Tight-binding forces bias harmonic and cubic force constants to larger lattice thermal conductivity values while maintaining the same directional ranking within each structure and relative alignment as in experiments, κ_latt_ (SnS) > κ_latt_ (SnSe) (see Figure S2). **Pnma**-SnS Quantum Espresso (QE) phonon dispersions compare in general well with the GFN-xTB results, the latter showing increased dispersion along U–R–*Z* (in agreement with ref (25)) but narrower dispersion above the phonon band gap. However, CP2K phonons are in general harder compared to QE and published phonon spectra.^23,25,47^ Forces were therefore mollified aiming at a better match of the acoustic CP2K band dispersions with QE results (based on SnS, see Figure S3), as acoustic frequencies significantly contribute to the lattice thermal conductivity.^48,49^ All xTB forces were accordingly scaled to 25% of their pristine value for all three structures considered in this work. The resulting SnS and SnSe phonon spectra are shown in Figure 3.

SnSe cell parameters were relaxed to *a* = 10.7111 Å, *b* = 3.9015 Å, and *c* = 3.9562 Å (experimental cell lengths are *a* = 11.501 Å, *b* = 4.153 Å, and *c* = 4.445 Å).^46^ For SnS, the difference between the *b-* and *c*-axis is less pronounced, while the layer stacking direction, *a*, is more compressed. The ratios *b*/*a* = 0.364 and *c*/*a* = 0.369 (experimental *b*/*a* = 0.361 and *c*/*a* = 0.386) indicate, for SnS, the compression of the accordion-like layers and reduction of the van der Waals interlayer spacing into a more isotropic equilibrium geometry compared to experiments. Phonon dispersions remain anisotropic though, with linear phonon band dispersion along Γ–*X* (*a*-axis), Γ–*Z* (*c-*axis), and Γ–*Y* (*b*-axis), in decreasing order of softness. Optical frequencies have markedly softened relatively to acoustic ones compared to SnS, an effect seen in other calculations^25^ and IR spectroscopy.^25,50^

### Lattice Thermal Conductivity

**Figure 4:**
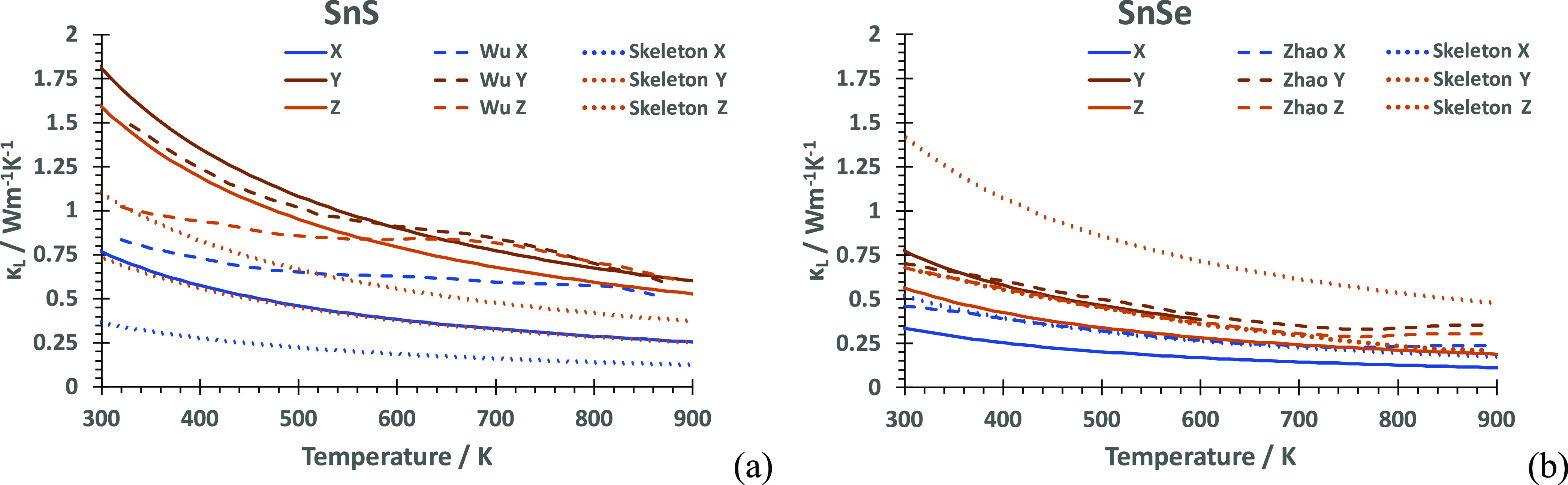
Lattice thermal conductivity, κ_latt_, for (a) SnS and (b) SnSe, resolved along Cartesian components as a function of temperature. Experimental measurements (SnS,^52^ SnSe^15^) and calculated curves (SnS,^23^ SnSe^17^) are shown for comparison.

The lattice thermal conductivities κ_latt_ of **Pnma**-SnS at 300 K are 0.7568, 1.8063, and 1.5776 W m^–1^ K^–1^ along the *a-*, *b-*, and *c-*axis, respectively, with an averaged isotropic lattice thermal conductivity of κ_iso_ = 1.3802 W m^–1^ K^–1^, in agreement with experimental values of 1.4^39^ and 1.25^51^ W m^–1^ K^–1^. These values correlate with the anisotropy of the structure and follow acoustic mode softness along Γ–*Y*, Γ–*Z*, and Γ–*X*, respectively. Overall, intralayer values are similar, while the lowest thermal conductivity is calculated along the layer stacking axis (Figure 4a). For **Pnma**-SnSe, the axis-resolved lattice thermal conductivities κ_latt_ at 300 K are 0.3374, 0.7726, and 0.5652 W m^–1^ K^–1^ for the *a*-, *b*-, and *c*-axis, respectively, with an averaged/isotropic lattice thermal conductivity κ_iso_ of 0.5584 W m^–1^ K^–1^. While the axial component sequence κ_b_ > κ_c_ > κ_a_ is the same order as for SnS, the anisotropy of lattice thermal transport is larger than that in SnS due to a more pronounced relative lowering of κ_c_ (Figure 4b). The calculated values at 300 K are in agreement with experimental values (0.46, 0.70, and 0.68 W m^–1^ K^–1^along the *a*-, *b-*, and *c-*axis,^15^ respectively), including their decrease to lowest values at high temperatures (<0.2 W m^–1^ K^–1^).

**Figure 5:**
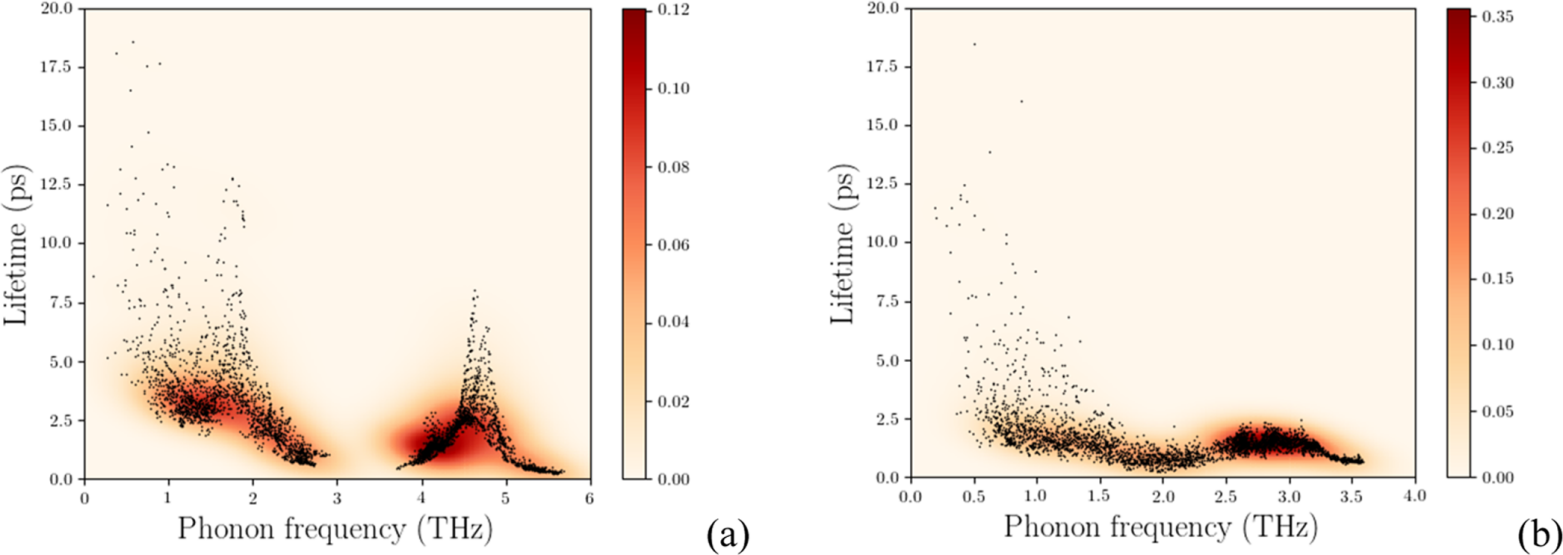
Phonon lifetimes versus frequency for (a) SnS and (b) SnSe. SnS is characterized by longer acoustic and optical lifetimes, the latter relax more rapidly in SnSe. A colormap underlines lifetime density differences.

SnS displays longer optical phonon relaxation times (>1.3 THz) than SnSe, for which sizable (>10 ps) relaxation times are found at lower frequencies only, see Figure 5. Optical frequency relaxation times are in general shorter in SnSe than those in SnS. Taken together, the loss of hotspot lifetime density from SnS to SnSe (Figure 5a,b) and the compactification of lifetimes in SnSe explain differences in calculated κ_latt_, indicating a broader impact over frequencies in the latter and not only a localized effect around lower/lowest frequencies. Furthermore, longer lifetimes at higher frequencies explain the more pronounced temperature effect on κ_a_ in SnS compared to SnSe, for which additional thermal conduction lowering is controlled by rapidly relaxing optical modes.

### Full Figure of Merit

In the single-lifetime approximation, the chemical potential μ was chosen to match the experimental Seebeck coefficients at a given temperature followed by τ fit on experimental electrical conductivity. In combination with lattice thermal conductivity, the full thermoelectric figure of merit *ZT* in SnS and SnSe can be evaluated and compared at different temperatures.

**Figure 6:**
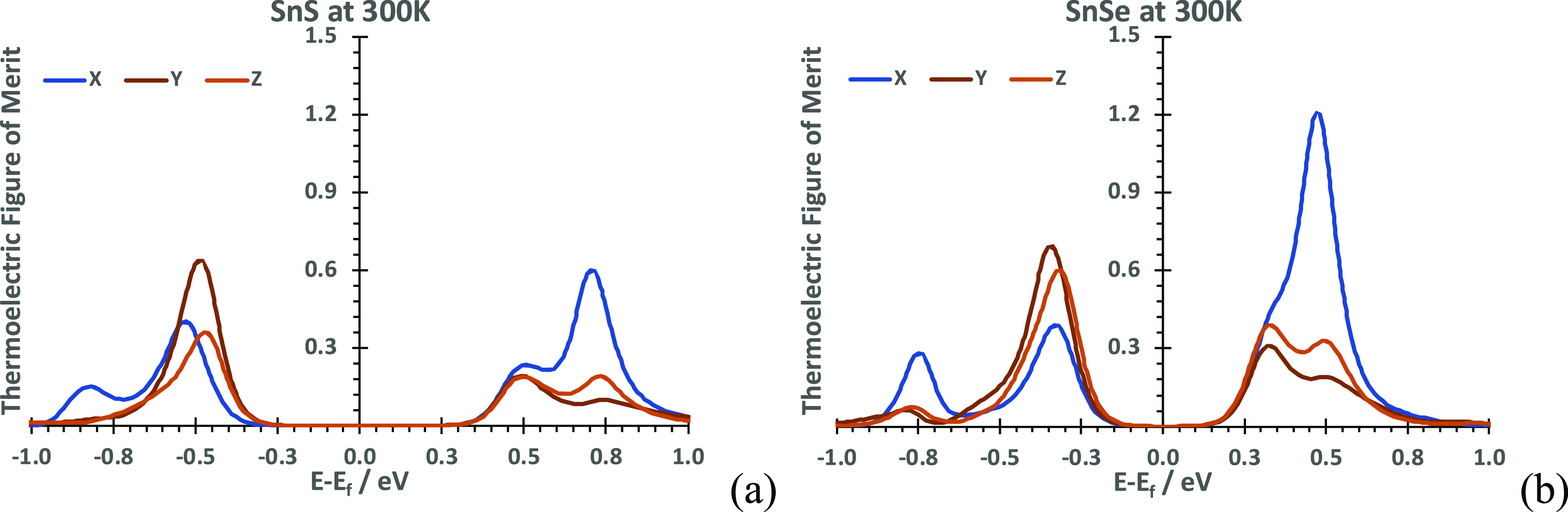
Axis-resolved thermoelectric figure of merits for (a) SnS and (b) SnSe, across the chemical potential range μ = ±1 eV.

The relaxation times at 300 K τ = 24.9394 fs (SnS) and τ = 20.1182 fs (SnSe) were calculated from alignment with experimental data (see Methods for details). Already at low temperatures, the transport anisotropy along different directions is apparent, with the *Y* direction (p-type) matching the *X* direction (n-type) in SnS, while in SnSe, the latter is larger (Figure 6). The similarity of the plots follows from the similarity in the band structure, with a narrower band gap and lower lattice conductivity in SnSe, making for higher maxima in both p and n regions.

**Figure 7:**
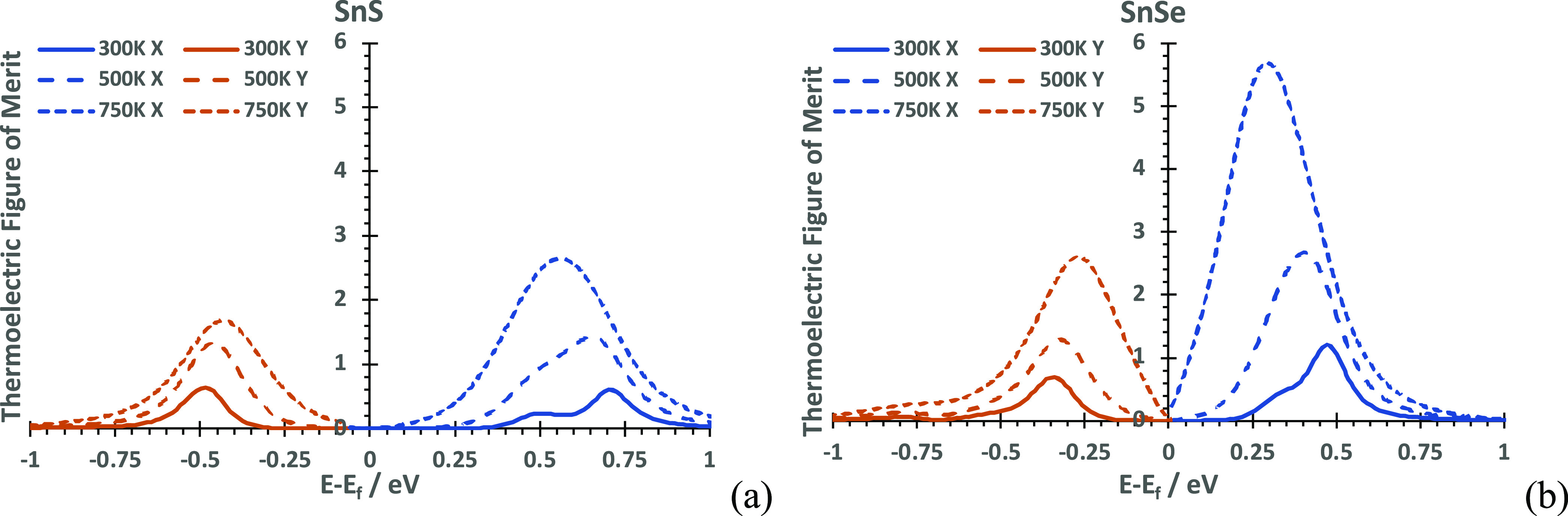
Thermoelectric figure of merits across chemical potentials at 300, 500, and 750 K for (a) SnS and (b) SnSe. The anisotropy between p and n regions increases as a function of temperature. Maxima for both compounds are found for the *X* (p regime) and *Y* (n regime) components.

For SnS, the relaxation times at 500 and 750 K are set to 12.3156 fs and 4.7640 fs, respectively. For SnSe, the values at the same temperatures are τ = 7.6324 and 5.7991 fs, respectively. Progression from 500 to 750 K promotes the dominance of n-type over p-type, in agreement with other calculations.^25^ The growth of the n-type maximum is noticeably more asymmetric in SnSe than that in SnS (Figure 7), confirming the important role of interlayer spacing and structural anisotropy. In comparison, the electronic-only thermoelectric figure of merit is less anisotropic (see Figure S4a,b), both among lattice directions and between n and p regimes, pinpointing the electronic similarity between SnS and SnSe.

## Comparison

Despite being structurally similar, **Pnma**-SnS is thermoelectrically inferior to **Pnma**-SnSe, due to higher thermal conductivity. As temperature increases, however, SnS becomes more similar to SnSe, with a steeper decrease in thermal lattice conductivity compared to SnSe (Figure 4). To better understand this effect, we have included a comparison with the high-temperature **Cmcm**-SnS phase. The latter results from compressing open zig-zag SnS layers (“accordion” geometry), achieving an additional in-layer Sn–S contact, accompanied by alternate layer antiparallel displacements. Its structural motif is not only relevant above the transition temperature, but it also plays a role of a “hidden” motif at lower temperatures through phonon modes that compress the accordion layers. We have therefore extended the comparison over the same temperature range as for the lower symmetry phases to deepen our understanding of how the structure–property relationship in this class of materials favors thermoelectricity. Thermal lattice calculations, geometry, and electronic structure for **Cmcm**-SnS are given in Section S5 and Figures S5–S8 in the SI.

### Lattice Thermal Conductivity

**Figure 8:**
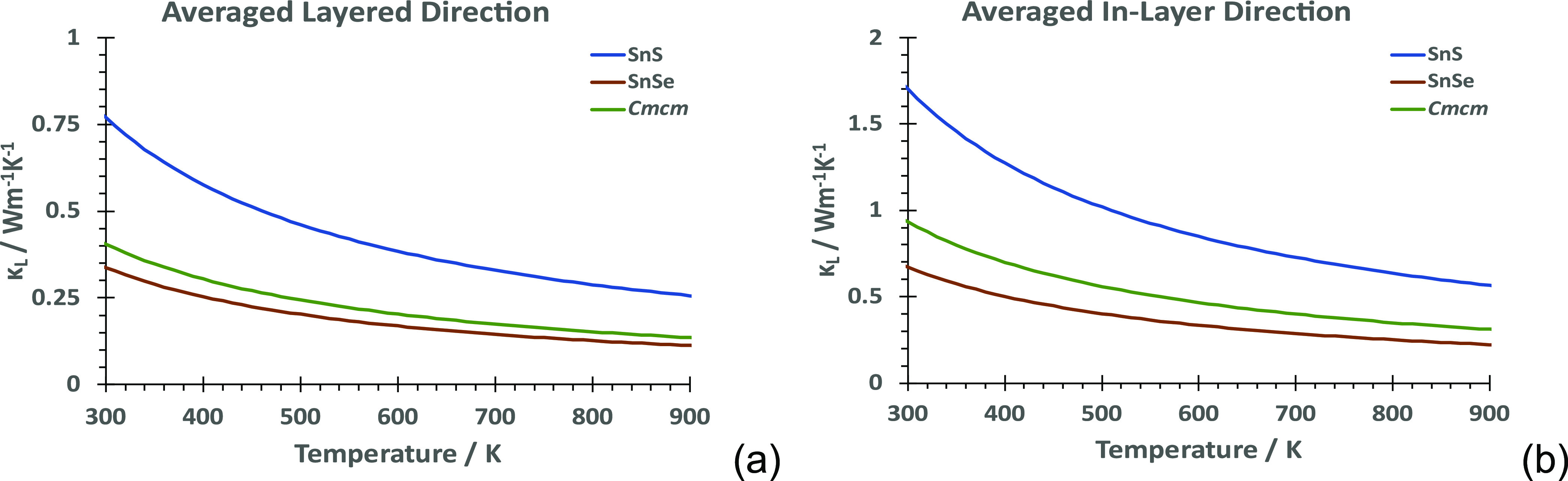
Lattice thermal conductivities between (a) the layered (layer stacking) direction (*a* in *Pnma*, *b* in *Cmcm*) and (b) the averaged intralayer direction (*b* and *c* in *Pnma*, *a* and *c* in *Cmcm*).

The direction-resolved lattice thermal conductivities are shown in Figure 8, separated into the cross-layer direction and the averaged intralayer direction. This highlights the difference between **Pnma**-SnS and **Pnma**-SnSe, with **Pnma**-SnSe less than half of **Pnma**-SnS across the temperature range. The **Cmcm**-SnS lattice thermal conductivity is closer to that of **Pnma**-SnSe than that of **Pnma**-SnS, indicating that structural similarities to **Cmcm** at higher temperatures are the reason for a better performance of **Pnma**-SnS. Anisotropy in κ_latt_ is due to different contributions to κ_latt_ along *a*, *b*, and *c*, particularly at lower frequencies (Figure S9). In **Cmcm**-SnS (Figure S9c) as in **Pnma**-SnS (Figure S9a), the contributions to κ_latt_ along *X*, and *Y*, *Z*, respectively, remain similar, while for SnSe, the contribution along *Z* is lower (Figure S9b).

**Figure 9:**
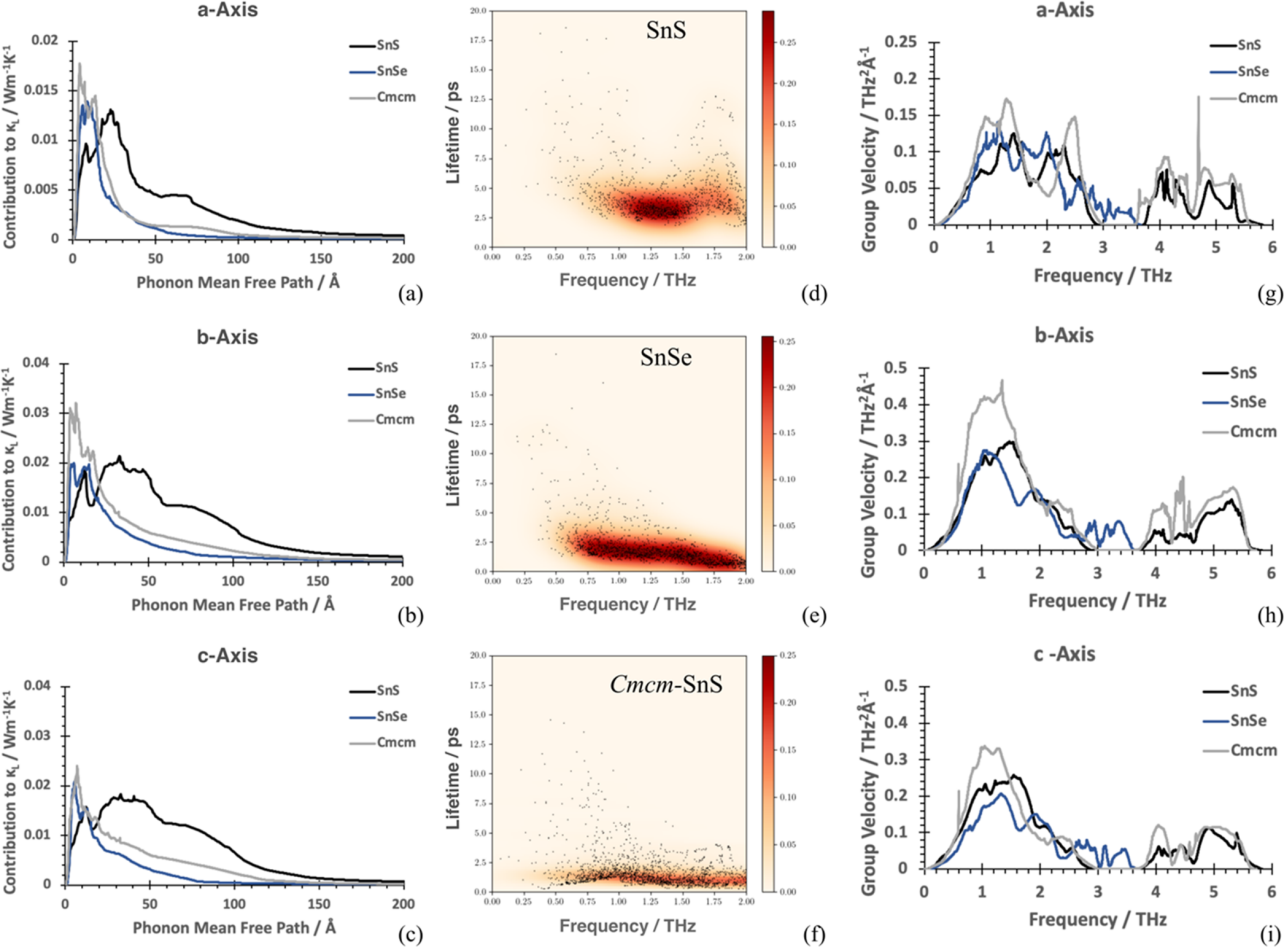
(a)–(c) Phonon contribution to the lattice thermal conductivity at 300 K versus phonon mean free path (MFP), (a) *Pnma*-a/*Cmcm*-b, (b) *Pnma*-b/*Cmcm*-c, and (c) *Pnma*-c/*Cmcm*-a. (d)–(f) Phonon lifetimes of *Pnma*-SnS, *Pnma*-SnSe, and *Cmcm*-SnS. The colormap refers to lifetime density. (g)–(i) Outer product of group velocities of phonon modes versus frequency: (g) *Pnma*-a/*Cmcm*-b, (h) *Pnma*-b/*Cmcm*-c, and (i) *Pnma*-c/*Cmcm*-a.

Like **Pnma**-SnSe, **Cmcm**-SnS shows faster optical mode relaxations compared to **Pnma**-SnS (Figure 9d–f). However, larger group velocities give rise to the increased lattice thermal conductivity of **Cmcm**-SnS (Figure 9g–i). Accordingly, similar lattice thermal conductivities across the layers in SnSe and **Cmcm**-SnS (Figure 8a) correspond to similar group velocity moduli (Figure 9g), while larger disparity in the lattice thermal conductivities along in-layer *b-* and *c-*axis are mirrored in group velocities differences along the same axes (Figure 9h,i).

The total group velocities for each structure across the entire frequency range, resolved into Cartesian directions *x*, *y*, and *z*, are 0.24303, 0.51925, and 0.46133 THz^2^/Angstrom for **Pnma**-SnS; 0.20491, 0.37799, and 0.30117 THz^2^/Angstrom for **Pnma**-SnSe; and 0.53466, 0.33741, and 0.75565 THz^2^/Angstrom for **Cmcm**-SnS.

The effect of larger group velocities by similar lifetimes leads to κ_latt_ contributions of mean free paths over a broader length range for **Cmcm**-SnS compared to **Pnma**-SnSe (see Figure 9a–c,g–i), while curve shapes are very similar in both compounds. Mean free path (MFP) contributions to κ_latt_ at shorter lengths (0–40 Å) account for most of κ_latt_ in **Pnma**-SnSe, while in **Pnma**-SnS, the distribution is broader, and the contribution is still sizeable above 40 Å (about 5 times larger than SnSe at 40 Å). By similar group velocity modules (Figure 9g–i), fast optical relaxation times in SnSe mainly account for this difference.

### Electronic Contribution and Figure of Merit

**Figure 10:**
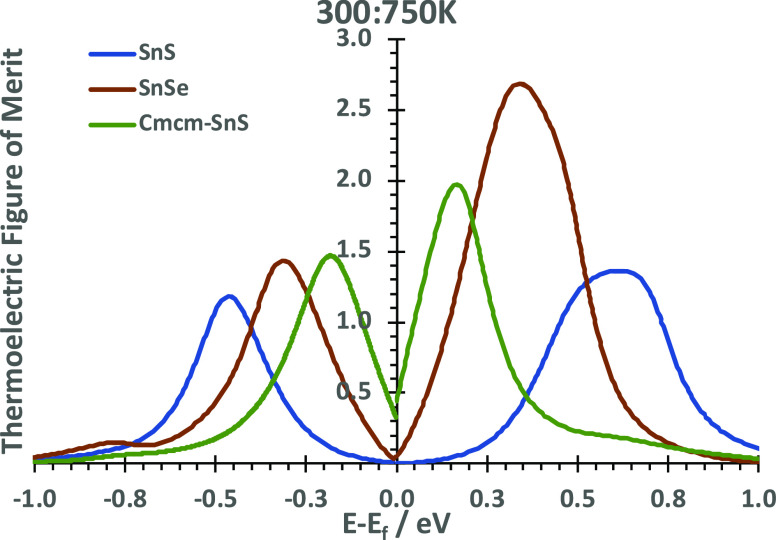
Averaged figure of merits between 300 and 750 K, across the chemical potential range μ ± 1 eV. Different structures are color-coded. For negative chemical potential values, the *X*-axis (*a* in SnS and SnSe, *b* in *Cmcm*-SnS) is shown, while for positive values, the *Y*-axis is used (*b* in SnS and SnSe, *c* in *Cmcm*-SnS).

To obtain an estimate of the impact of electronic transport anisotropy over a relevant temperature range (Figure 10), we show the figure of merit *ZT* versus chemical potential, obtained by averaging values at 300, 500, and 750 K. *ZT* at individual temperatures are given in Figure S10. For negative μ, maxima are along the in-layer *b* direction (*c* in **Cmcm**), while the layer stacking direction *a* (*b* in *Cmcm*) affects maxima in the positive range.

## Discussion

The ranking of calculated thermal conductivities for **Pnma**-SnS and -SnSe is in good agreement with experimental results. Inclusion of three-phonon scattering and its effect on lifetimes (Figure 9) seem therefore to account for the relevant physics, while isotope effects can be neglected as discussed elsewhere.^23^ This is also in agreement with frequency lowering due to the larger Se mass and Sn–Se chemical bond softening.^25^

Lower frequency phonon modes are major heat carriers as illustrated by the contribution to lattice thermal conductivity by frequency, with acoustic phonons becoming dominant from SnS to SnSe, as noticed above. Interestingly, the **Cmcm**-SnS phase appears closer to SnSe, pinpointing the central role of in-layer dynamics in reducing finite lifetimes, especially of lower optical frequencies.

**Pnma**-SnSe is characterized by lifetimes in the low-frequency region that are lower than **Pnma**-SnS, also reflected by shorter mean free path (MFP) contributions in **Pnma**-SnSe compared to **Pnma**-SnS. Compactification of MFP contributions to lower frequencies is also present in **Cmcm**-SnS, which, however, remains above SnSe values, due to larger group velocities in the **Cmcm*-*SnS phase, in particular along the layering *a* direction. This entails the largest difference in lattice thermal conductivity between **Pnma**-SnSe and **Cmcm**-SnS. We argue that this is a consequence of symmetry and that temperatures just below phase transition would allow for group velocity rescaling, an effect that could be verified with lattice dynamics calculations.

From the comparison of lattice thermal conductivities, phonon lifetimes, and mean free paths, phonon scattering near and across the transformation from **Pnma** to **Cmcm** appears key to thermoelectricity in this class of materials, while high symmetry seems to enforce higher phonon group velocities. Accordingly, **Pnma**-SnS turns into a better thermoelectric, as it becomes similar to *Cmcm* on increasing the temperature, as shown in this work. The increased importance of **Cmcm** at temperatures below the phase transition (*T* = 500 K) is illustrated in Figure S16 by isothermal–isobaric molecular dynamics simulations. In doing so, SnS becomes at the same time more like SnSe, thanks to in-layer deformation phonon softening.

Besides low lattice thermal conductivity, thermoelectric materials must be robust in their “electronic suitability.” While **Pnma**-SnS and -SnSe are electronically closer than their lattice conductivities differ (Figures S11 and S12), the former is superior in its Seebeck coefficient, while electrical conductivity is larger for SnSe. The *a*-axis power factors across the chemical potential range are very similar, whereas in-layer (*b-* and *c*-axis) values are larger for **Pnma**-SnS. Therefore, the main reason for the increase in the overall figure of merit of **Pnma**-SnSe is related to a larger decrease in lattice thermal conductivity. For **Cmcm**-SnS (Figure S13), the low Seebeck is balanced by comparatively high electronic conductivities. Even if the interlayer (*a*-axis) power factor favors n-type and in-layer (*b*- and *c*-axis) favors p-type, the difference is less pronounced than that for the **Pnma** phase. This confirms that the role of **Cmcm** in lowering thermal transport lies mainly in its lattice dynamical influence, with electronics only moderately affecting its thermoelectric properties.

The maximal figure of merits *ZT*s for SnS and SnSe entail an increase in the layered direction as an n-type, whereas most experimental measures focus on in-layer p-type thermoelectrics, with values similar to the p-type figure of merits calculated in this paper. As the n-type maxima are obtained at larger chemical potentials, this would imply a further increase in the level of doping in real materials as a route to maximizing *ZT* beyond current reports.

## Conclusions

In this work, we have calculated thermal end electronic transport for SnS and SnSe, using the frozen phonon and single relaxation time approximations. Phonons were calculated based on a transferable tight-binding (xTB^41^) potential, which resulted in a reliable comparison with experiments, including the relative ranking of SnS and SnSe, anisotropy, and absolute lattice conductivity values. This improvement stems from larger supercells and from the xTB potential correctly capturing frequency softening at higher temperatures. Full figures of merit were obtained by including the electronic part via the Boltzmann transport equation based on the Wannier function interpolation of the electronic bands and single relaxation time, showing asymmetry between p-type and n-type regimes.

The lower thermal conductivity of SnSe compared to SnS is related to shorter lifetimes of optical modes, besides softer acoustic frequencies in SnSe. The inclusion of a complete analysis of the **Cmcm** high-temperature SnS phase suggests that this softening should be associated with in-layer *b*, *c* moduli compression, which indicates a dynamical role of a hidden **Cmcm** phase already at temperatures below phase transition. Once formed, **Cmcm**-SnS shows larger group velocities in the in-layer components, an intrinsic effect that contributes to preventing lattice thermal conductivity to reach even lower values.

Taken together, our findings provide a reliable, efficient, and scalable scheme to calculate thermal conductivity in tin chalcogenides, one that can be transferred to larger unit cells for composite material design and nanoinclusions. In combination with electronic transport calculations based on Wannier functions, a robust scheme for *ZT* thermoelectric rankings can be achieved, broadly suitable for thermoelectric material characterization.

Our analysis suggests that **Pnma**-SnS does become a better thermoelectric at higher temperatures, as it resembles more and more **Cmcm**-SnS. Dynamical in-layer compression is indicated as a key mechanistic element beyond thermal conductivity lowering, already before the **Cmcm**-SnS phase fully locks in. Leveraging this effect at lower temperatures is therefore key to engineering better thermoelectric materials based, here, on tin chalcogenides.

## Supporting Information Available

SnS and SnSe tight-binding (GFN-xTB) relaxed geometries; lattice thermal conductivity (pristine GFN-xTB forces); SnS phonons and comparison between QE and CP2K/xT; electronic figure of merit; full characterization of *Cmcm*-SnS (structure optimization and bands, phonons, thermal conductivity and phonon lifetimes, full figure of merit); comparison among SnS, SnSe, and *Cmcm*-SnS (contribution to κ_latt_ as a function of frequency, *ZT* as a function of chemical potential, theoretical operational efficiency (TOE), and electronic transport calculations (Boltzwann)); comparison of RTA and LBTE results; convergence tests (lattice thermal conductivity and lattice thermal conductivities as a function of q-mesh size); and SnS: molecular dynamics simulations (PDF)
