# Supplementary material for: Layered Tin Chalcogenides SnS and SnSe: Lattice Thermal Conductivity Benchmarks and Thermoelectric Figure of Merit
Source: J Phys Chem C Nanomater Interfaces. 2022 Aug 16;126(33):14036–46. doi: 10.1021/acs.jpcc.2c02401 (PMC9421910; doi:10.1021/acs.jpcc.2c02401)
Supplement: Supplementary file 1 — jp2c02401_si_001.pdf [file jp2c02401_si_001.pdf]

Supplementary Information

Layered Tin Chalcogenides SnS and SnSe: Lattice Thermal Conductivity Benchmarks and Thermoelectric Figure of Merit

Jordan Rundle[1] and Stefano Leoni[1,*]

[1] Materials Discovery Group, School of Chemistry, Cardiff University, C10 3AT Cardiff, UK

[*] Electronic Address: leonis@cf.ac.uk

Table of Contents

S1. SnS and SnSe tight-binding (GFN-xTB) relaxed geometries ***3***

S2. Lattice thermal conductivity, pristine forces ***3***

S3. SnS Phonons: QE and CP2K/xTB ***3***

S4. Electronic figure of merit ***4***

S5. Full characterisation of Cmcm-SnS. ***4***

S5.1 structure optimisation and bands **4**

S5.2 Phonons **5**

S5.3 Thermal conductivity and phonon lifetimes **5**

S5.4 Full figure of merit **6**

S6. Comparison ***7***

S6.1 Contribution to κ_{latt} as a function of frequency **7**

S6.2 ZT as a function of chemical potential **8**

S6.2.1 Theoretical operational efficiency (TOE) **8**

S6.3 Electronic transport calculations (Boltzwann) **9**

S6.3.1 SnS 9

S6.3.2 SnSe 10

S6.3.3 *Cmcm*-SnS 11

S7. Comparison of RTA and LBTE results ***12***

S8. Convergence tests ***13***

S8.1 Convergence of lattice thermal conductivity **13**

S8.2 Convergence of lattice thermal conductivities as a function of q-mesh size **14**

S9. SnS: Molecular Dynamics simulations ***15***

S10. References ***17***

S1. SnS and SnSe tight-binding (GFN-xTB) relaxed geometries

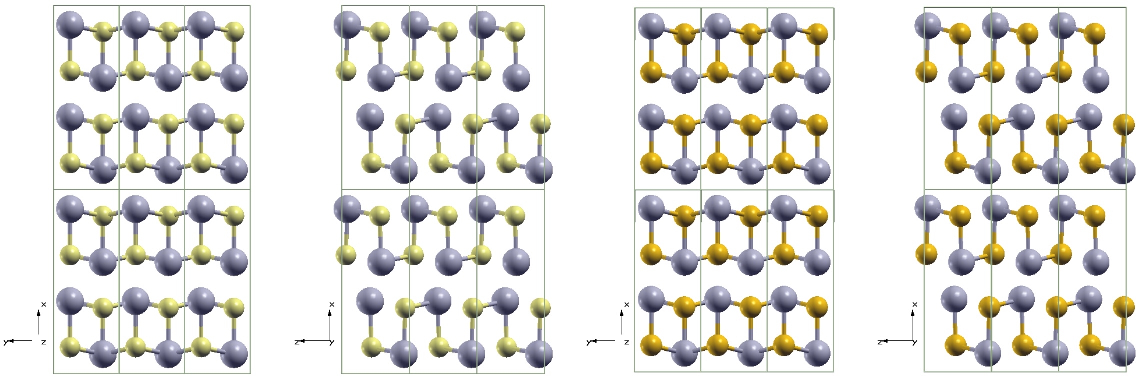

Figure S1: Tight-binding (xTB) equilibrium geometries for SnS and SnSe, calculated using CP2K, showing a compression of the zigzag, “accordion-like” layers along Z and a reduction of interlayer spacing along X. Space group after relaxation is *Pnma*. Sn is gray, S yellow and Se orange. The standard 8-atoms unit cell is indicated.

S2. Lattice thermal conductivity, pristine forces

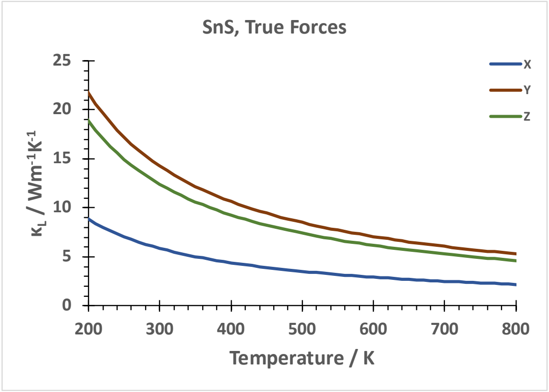

(a)

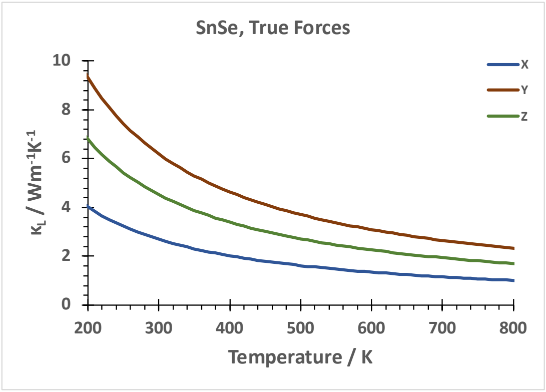

(b)

Figure S2 Lattice thermal conductivity, κ_{latt}, resolved into its X Y and Z components (corresponding to *a*, *b* and *c* axes) as a function of temperature. For (a) SnS, Y and Z components are similar, while for (b) SnSe, Y keeps an intermediate value.

S3. SnS Phonons: QE and CP2K/xTB

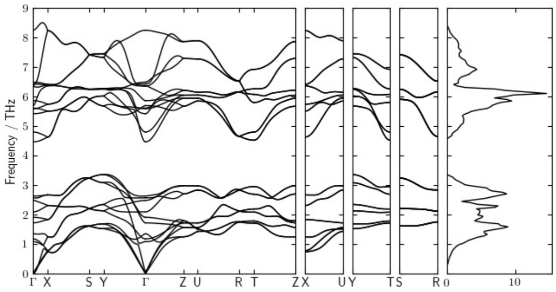

(a)

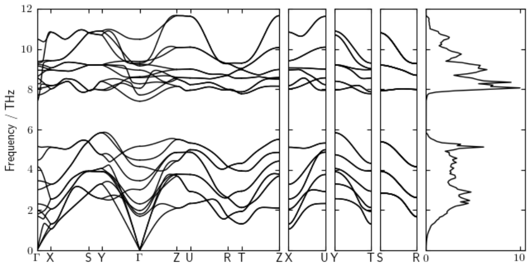

(b)

Figure S3 (a) Quantum Espresso and (b) CP2K phonon spectra for SnS. In (b), pristine xTB forces were used.

S4. Electronic figure of merit

The electronic figure of merit is expressed as $ZT_e = S^2\sigma/\kappa_e$.

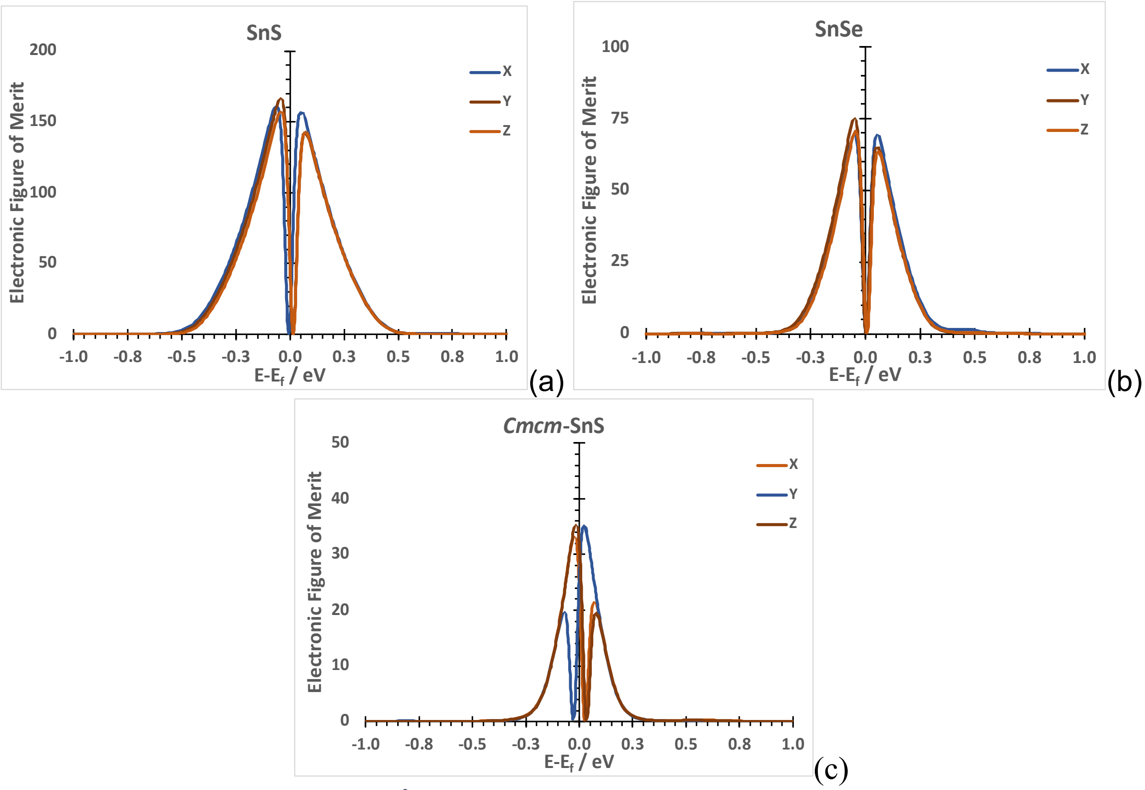

Figure S4 Electronic figure of merit $ZT_e = S^2\sigma/\kappa_e$, for (a) *Pnma*-SnS, (b) *Pnma*-SnSe and (c) *Cmcm*-SnS. *Pnma*-SnS has the largest ZT_e, owing to its larger Seebeck coefficient (see below, Figures S11-S13). ZT_e X, Y and Z components are shown in blue, brown and orange, respectively.

S5. Full characterisation of *Cmcm*-SnS

S5.1 structure optimisation and bands

The *Cmcm*-SnS primitive unit cell was relaxed (Quantum Espresso) into $a = b = 6.2607$ Å, $c = 4.1207$ Å, $\beta = 141.592$° (orthorhombic *C*-cell: $a = 4.1188$ Å, $b = 11.8247$ Å and $c = 4.1207$ Å). These values are comparable to experiments ($a = 4.177$ Å, $b = 11.480$ Å and $c = 4.148$ Å [S1], setting adapted) and calculations ($a = b = 6.208$ Å, $c = 4.108$ Å, $\beta = 141.322$°) [S2]. The xTB relaxed structure had $a = 3.9774$ Å, $b = 10.1644$ Å and $c = 3.5628$ Å. As for *Pnma* structures, xTB yields a more compact structure and denser layer packing along the layer-stacking direction b (corresponding to a in *Pnma*).

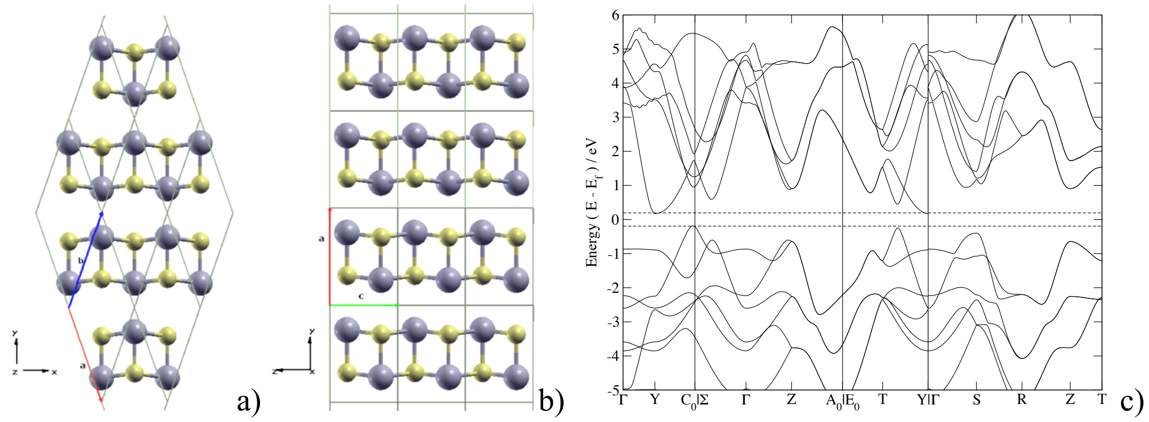

Figure S5 Relaxed *Cmcm*-SnS structure (QE); (a) primitive cell, (b) *C*-cantered orthorhombic cell, (c) *Cmcm*-SnS GGA-PBE band structure calculated along high-symmetry k points. Sn is grey, S is yellow. Crystallographic axes are indicated for both settings, as well as Cartesian axes used to calculate transport coefficients.

The band structure shows a direct band gap of 0.706 eV and an indirect band gap of 0.381 eV (Literature: indirect band gap 0.3 eV [S3], 0.42 eV [S4] and 0.7 eV [S5] LDA, experimental lattice parameters). The valence band maximum (VBM) is at Σ_0 and the conduction band minimum (CBm) at Y.

S5.2 Phonons

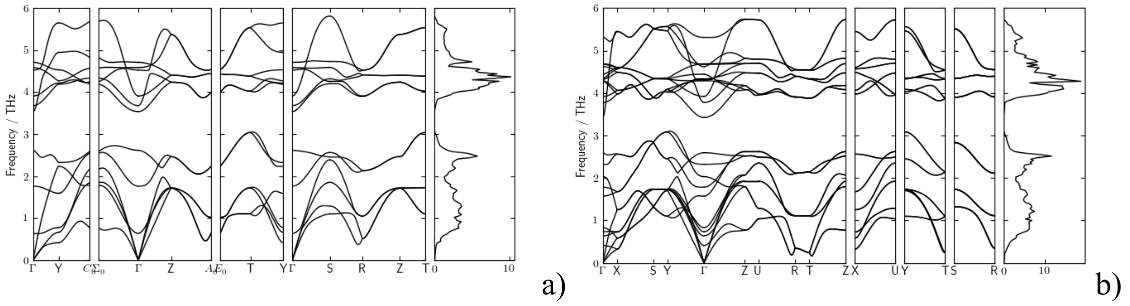

Figure S6 Phonon dispersion spectra for *Cmcm*-SnS obtained with PHONOPY, using CP2K as force driver based in the xTB potential; (a) primitive cell, (b) orthorhombic *C*-centered cell.

S5.3 Thermal conductivity and phonon lifetimes

The supercell sizes chosen for the calculation of the lattice thermal conductivity were 3x3x3 for the third order interactions and 6x6x6 for the second order interactions. The lattice thermal conductivity of *Cmcm*-SnS at 300K is 0.8308, 0.4049, and 1.0338 $Wm^{-1}K^{-1}$, for the x, y and z directions respectively (corresponding to *Pnma c*, *a* and *b* axes) with an isotropic average of 0.7565 $Wm^{-1}K^{-1}$. At 850 K, the lattice thermal conductivity of *Cmcm*-SnS is 0.2929, 0.1436, and 0.3651 $Wm^{-1}K^{-1}$, with an isotropic average of 0.2672 $Wm^{-1}K^{-1}$. This value is smaller

than in experiments, with the lattice thermal conductivity of *Cmcm*-SnS usually measured at 0.5 $Wm^{-1}K^{-1}$[S4, S6], but shows the expected trend of temperature dependence and direction anisotropy.

Cmcm-SnS exhibits sharp reduction in phonon frequency lifetimes compared to *Pnma*-SnS, especially in the low-frequency region. A significant shortening of optical modes lifetimes is visible over 1.3 THz, similar to what was noticed for SnSe.

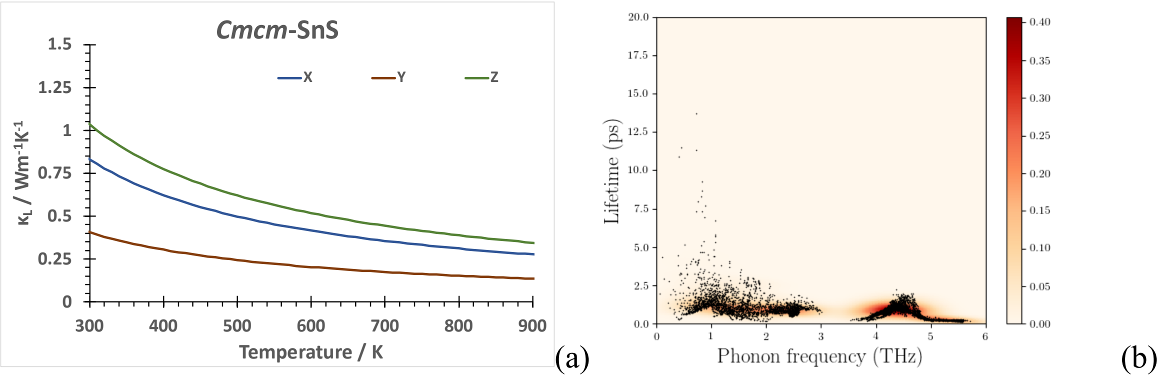

Figure S7 (a) Lattice thermal conductivity, κ_{latt}, along the X, Y and Z directions as a function of temperature; (b) phonon lifetimes vs. frequency. The heatmap refers to lifetime densities.

S5.4 Full figure of merit

The *Cmcm* phase of SnS is experimentally not stable in the lower temperature range, therefore the same relaxation times as *Pnma*-SnS were used. Here we evaluate its figure of merit outside its stability range to gain a deeper understanding on the structure-property relationship in this material. Due to the reduced bandgap and lower κ_{latt} *Cmcm*-SnS shows similarities in the p- and n- maxima with *Pnma*-SnSe, while numerically, maxima are closer to *Pnma*-SnS.

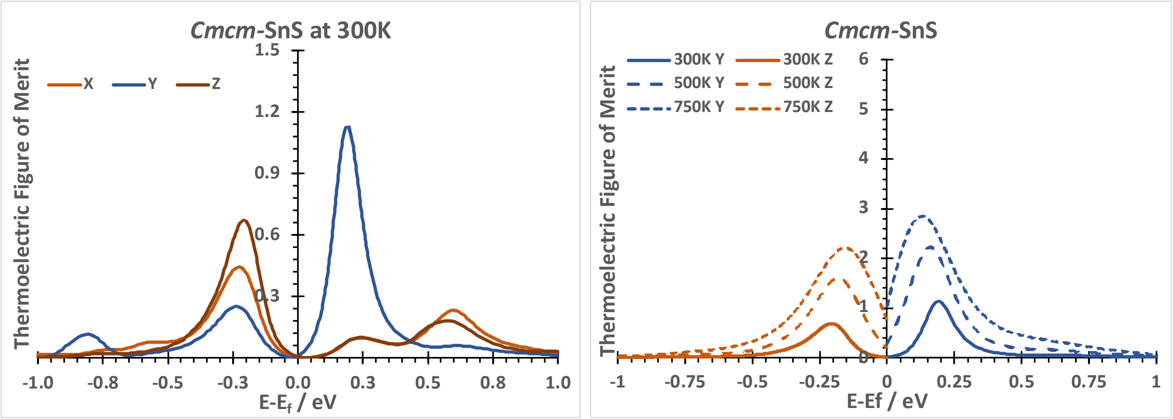

Figure S8 Figure of merits vs. chemical potential at 300, 500 and 750 K. Y axis values are larger below the Fermi level while X axis values dominate above.

S6. Comparison

S6.1 Contribution to κ_{latt} as a function of frequency

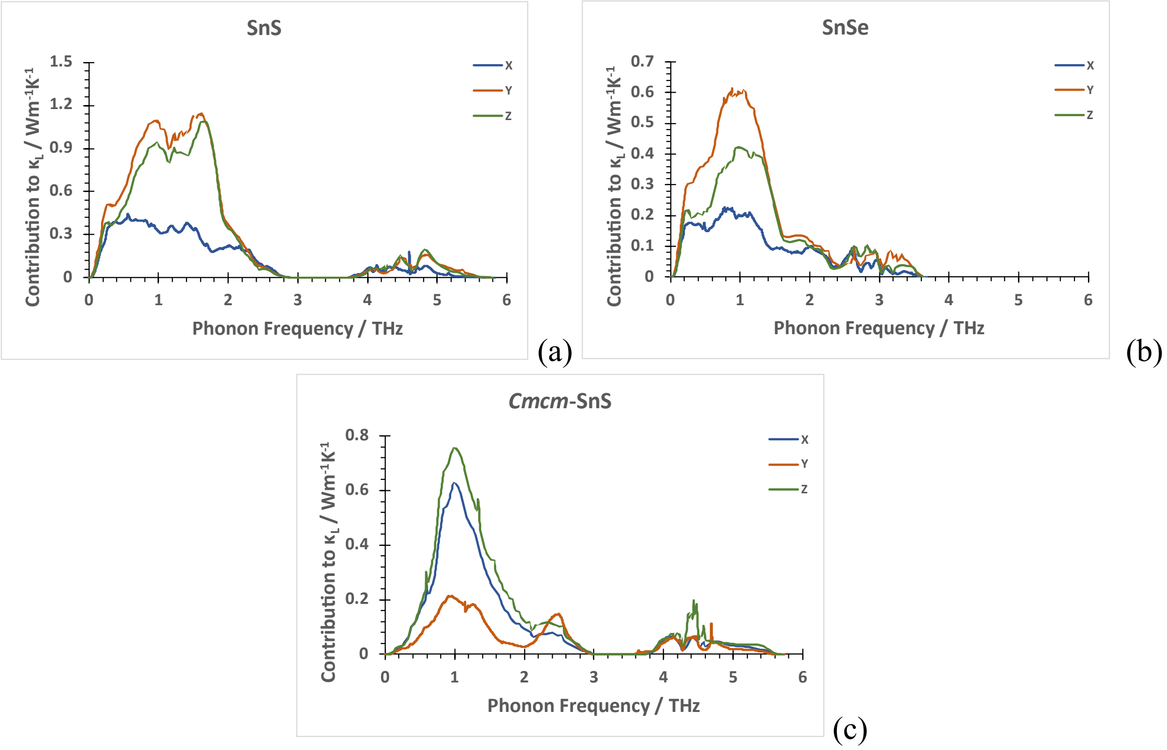

Figure S9 Contributions to lattice thermal conductivity by phonon frequency for (a) SnS, (b) SnSe and (c) *Cmcm*-SnS. Anisotropy is due to differences at low frequencies, with X (*a* axis in SnS, SnSe) and Y (*b* axis in Cmcm-SnS) smaller compared to the other directions. In SnSe, *b* and *c* directions (Y and Z) are less alike. In *Cmcm*, X and Z directions stay similar, an effect that can be attributed to group velocities moduli.

S6.2 ZT as a function of chemical potential

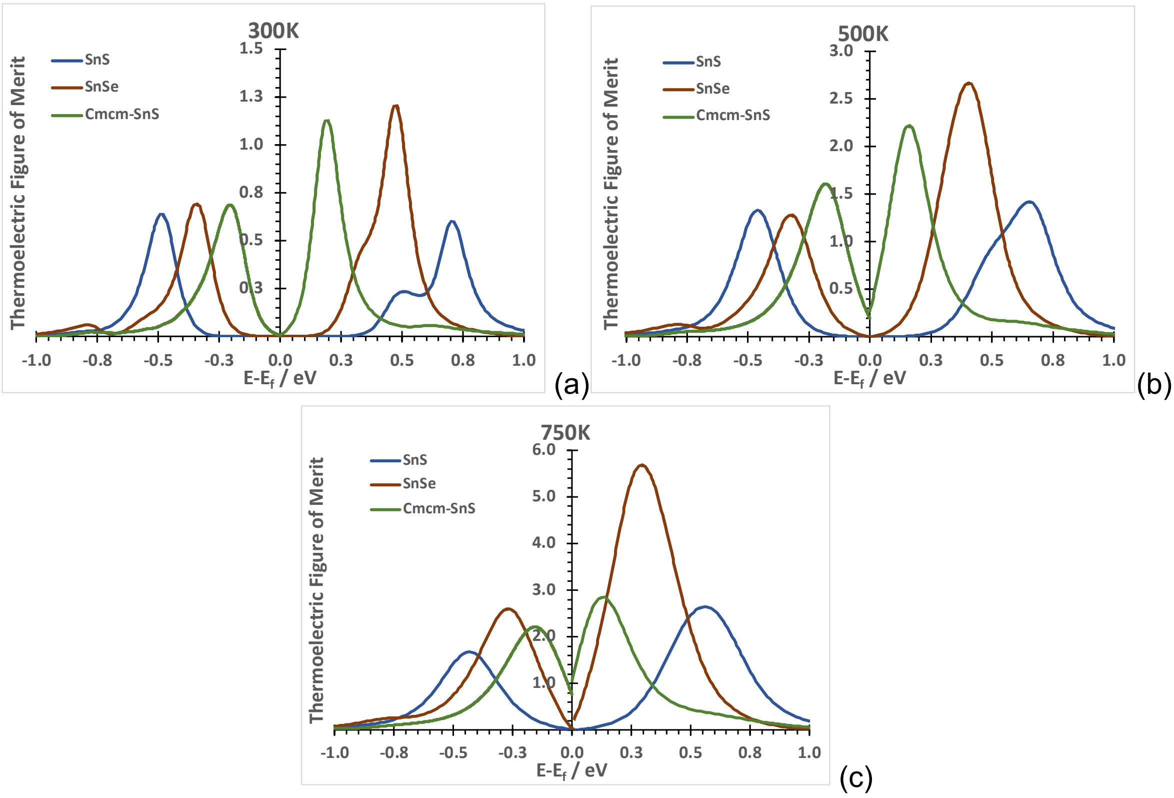

Figure S10 Full figure of merit of SnS, SnSe and *Cmcm*-SnS at (a) 300 K, (b) 500 K and (c) 750 K. A hypothetical low-temperature *Cmcm* phase would have a stronger figure of merit than its corresponding Pnma structure. This difference narrows on increasing temperature, due to *Pnma*-SnS becoming more similar to *Cmcm*-SnS.

S6.2.1 Theoretical operational efficiency (TOE)

The value of ZT directly influences the efficiency under operational conditions of thermoelectric according to [S7]:

$$\epsilon = \frac{T_H - T_C}{T_H} \frac{\sqrt{1+ZT} - 1}{\sqrt{1+ZT} + \frac{T_C}{T_H}}$$

TOE:

SnS (8.30% T=500K, 17.98%, T=750K);
SnSe (12.35% T=500K and 25.67% T=750K);
Carnot limit (40% T=500K and 60% T=750K).

S6.3 Electronic transport calculations (Boltzwann)

S6.3.1 SnS

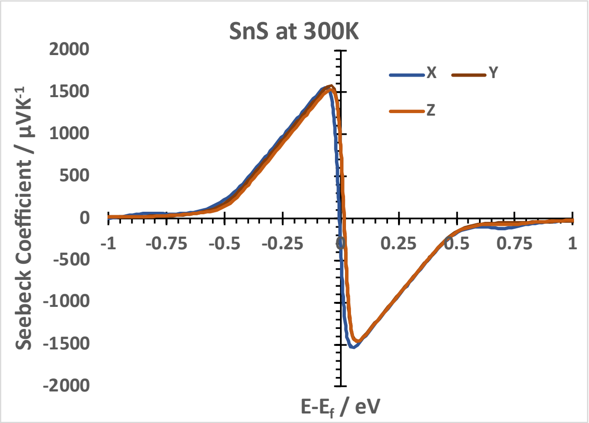

(a)

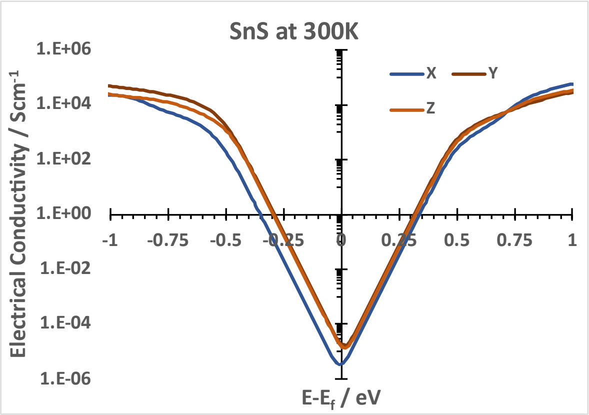

(b)

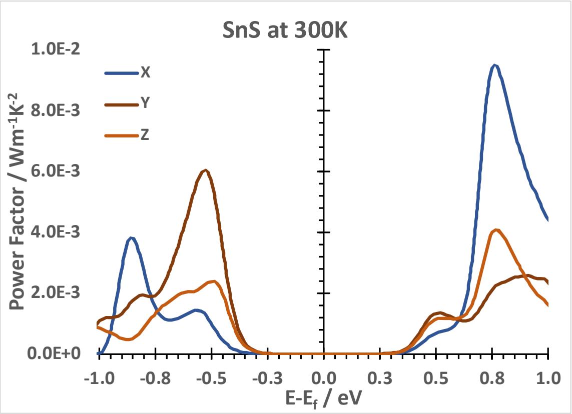

(c)

Figure S11 (a) Seebeck coefficient, (b) electrical conductivity and (c) Power factor vs chemical potential at 300 K. The power factor is defined as $PF = \sigma S^2$.

S6.3.2 SnSe

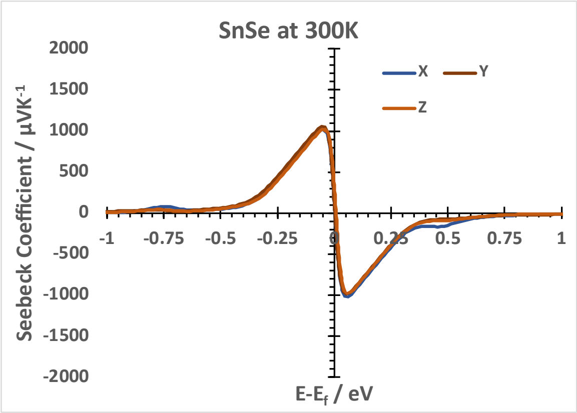

(a)

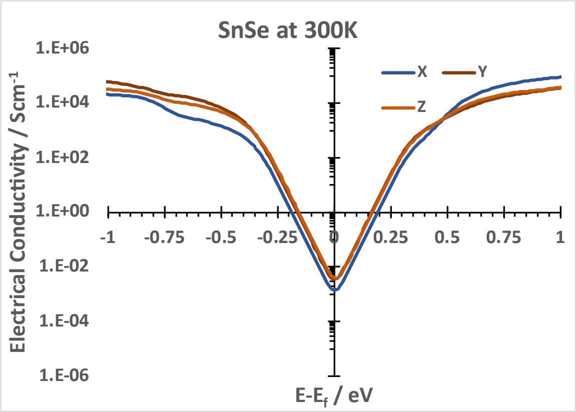

(b)

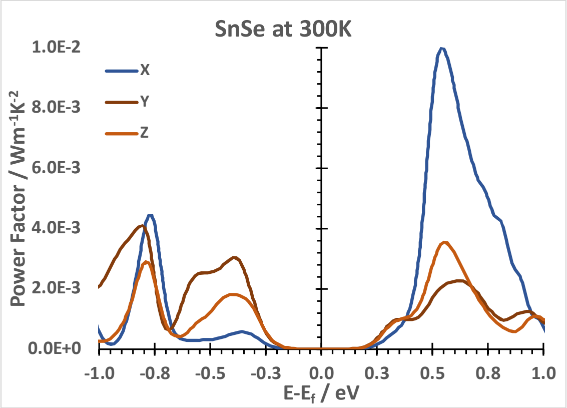

(c)

Figure S12 (a) Seebeck coefficient, (b) electrical conductivity and (c) Power factor vs chemical potential for SnSe, at 300 K. The power factor is defined as $PF = \sigma S^2$.

S6.3.3 *Cmcm*-SnS

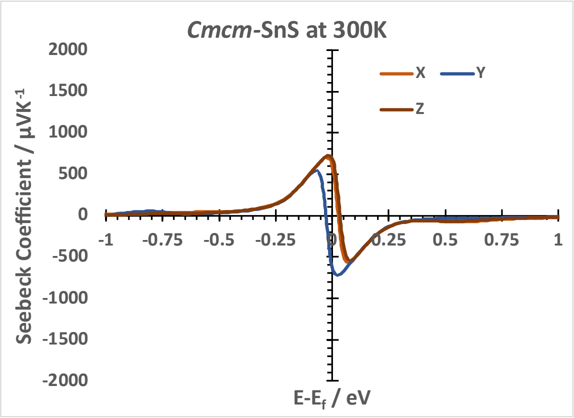

(a)

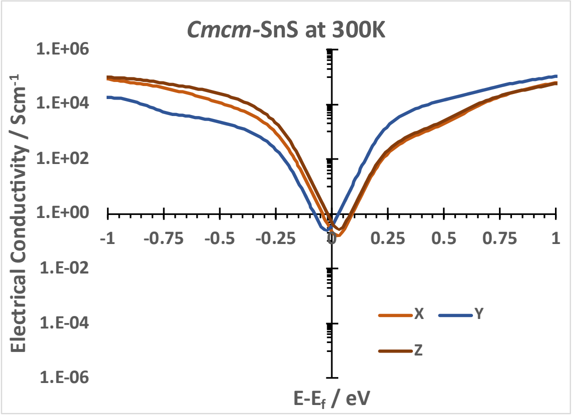

(b)

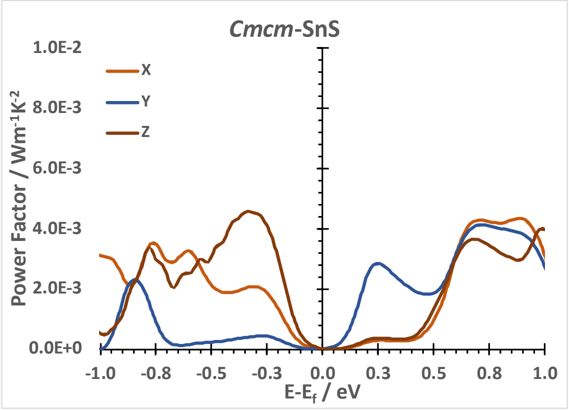

(c)

Figure S13 (a) Seebeck coefficient, (b) electrical conductivity and (c) Power factor vs chemical potential for *Cmcm*-SnS, at 300 K. The power factor is defined as $PF = \sigma S^2$.

S7. Comparison of RTA and LBTE results

Table S1 SnS: RTA/LBTE comparison at different temperatures. Sample mesh size, 5x13x13. Cutoff pair distance = 15 Å (39.13%).

300	RTA	0.7353	1.7731	1.5444
	LBTE	0.7407	1.8971	1.7015
500	RTA	0.4412	1.0621	0.9254
	LBTE	0.4443	1.1357	1.0188
800	RTA	0.2757	0.6635	0.5781
	LBTE	0.2777	0.7093	0.6363

Table S2 SnSe: RTA/LBTE comparison at different temperatures. Sample mesh size 5x13x13.

300	RTA	0.3275	0.7700	0.5545
	LBTE	0.3223	0.8048	0.5859
500	RTA	0.1966	0.4618	0.3327
	LBTE	0.1934	0.4827	0.3515
750	RTA	0.1311	0.3079	0.2218
	LBTE	0.1290	0.3217	0.2343

S8. Convergence tests

S8.1 Convergence of lattice thermal conductivity

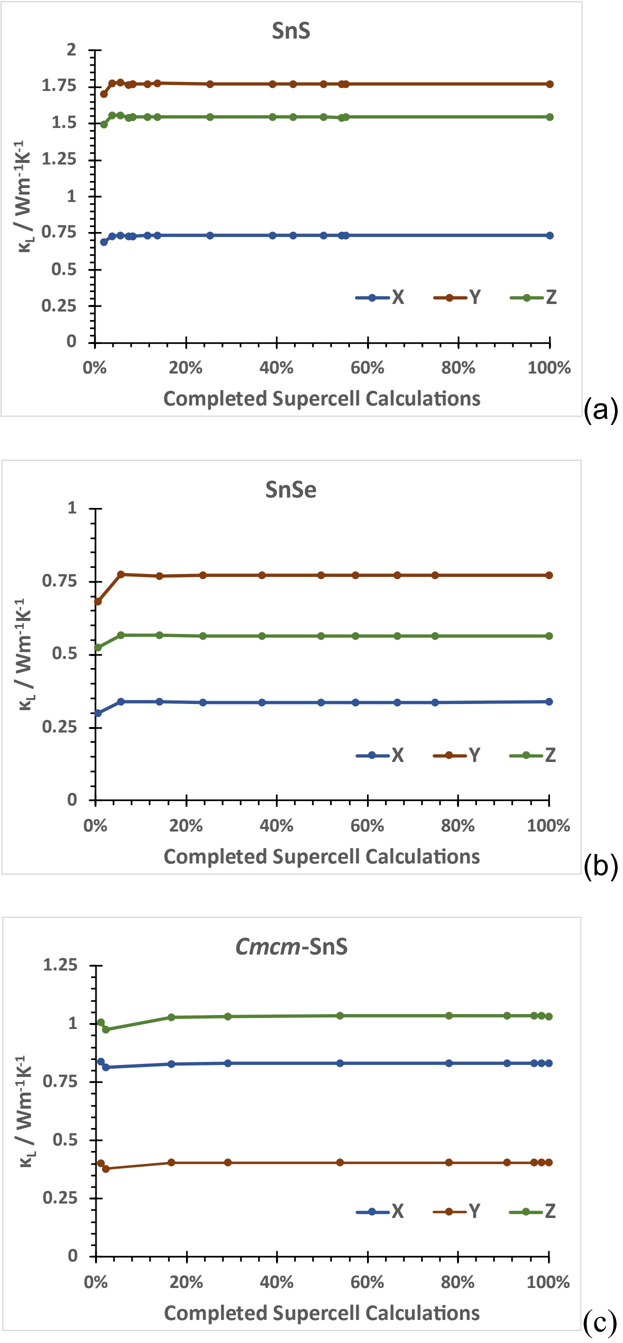

Figure S14 Convergence of lattice thermal conductivity at increasing cutoff pair distance values, shown as a percentage of the full number of calculations without cutoff.

S8.2 Convergence of lattice thermal conductivities as a function of q-mesh size

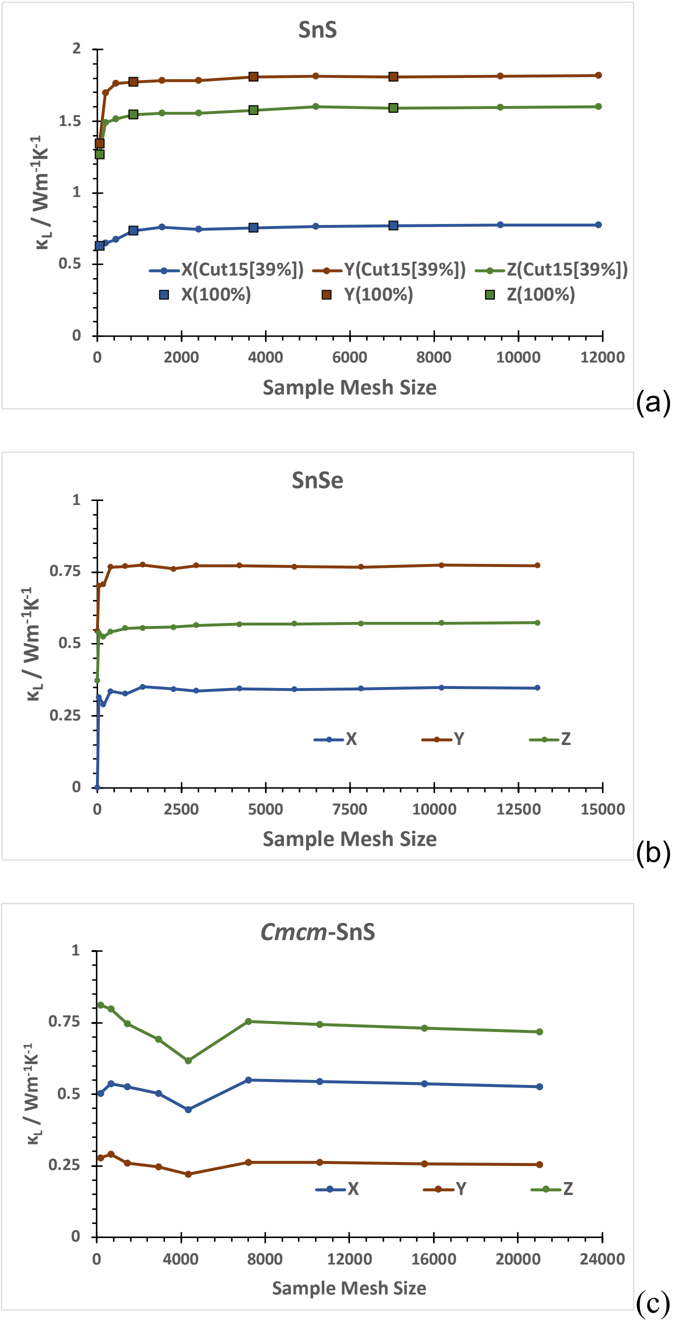

Figure S15 Convergence of the lattice thermal conductivities at increasing sampling mesh sizes, where the size is the number of grid points defined by $N = N_x \times N_y \times N_z$.

S9. SnS: Molecular Dynamics simulations

Isothermal (T=500 K), isobaric (p=1 bar) molecular dynamics simulations were performed on SnS *Pnma*, interatomic forces were calculated as for lattice thermal transport calculations. The Newton's equations of motions were integrated using a velocity Verlet scheme, the timestep was 0.2 fs. Trajectories were analyzed based on the coordination number (CN) of Sn by S. The occurrence of *Cmcm* motifs is associated with coordination number (CN=7), which is characteristic of the α-TlI type (*Cmcm*) structure. In Fig S16, snapshots *b*, *d* and *f* correspond to regions of high *Cmcm* content (~50%, ~60% and ~50%, respectively), while in *a*, *c* and *e* only the modes that squeeze the accordion-like SnS layers are active. All in all, while *Cmcm* cannot fully lock-in due to thermodynamics, its structural motifs and the soft modes associated with the phase transition are nonetheless already visible and manifest themselves in the frequent occurrences of CN=7. Further details are in the caption of Fig. S16.

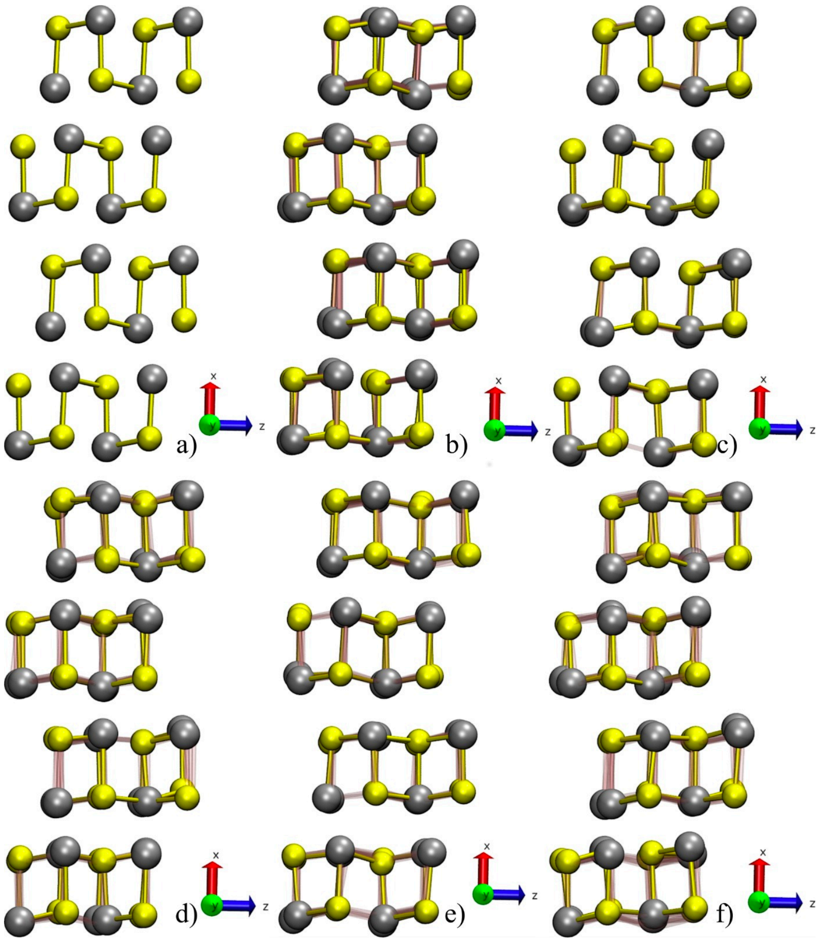

Figure S16 Snapshots of a representative molecular dynamics simulation trajectory of SnS at T=500 K. The coordination number (CN) of Sn by S was used to select snapshots and identify *Cmcm* phase growth attempts. A CN=7 would signal the occurrence of the α-TlI type structure of the high-temperature *Cmcm* phase. a) t=0 ps, SnS *Pnma*; b) 0.6 ps, 50% Sn have CN=7. Bonds are averaged around this configuration for +/- 0.1 ps (transparent brown); c) 1 ps, in-plane compression of the

Pnma layers; d) 1.5 ps, 60 % Sn have CN=7. On the average the system is closer to *Cmcm* (brown transparent bonds); e) 1.9 ps all connections within layers are shorter, individual layers are as in *Cmcm* but overall layers are misaligned; f) 2.3 ps, the *Cmcm*/α-TlI motif is clearly visible, both in the in-layer compression and the relative interlayer shearing. Same bond averaging as for b) and d).

S10. References

[S1] H. Wiedemeier and F. J. Csillag, Z. Kristallogr. **149**,17, (1979).

[S2] Materials Data on SnS by Materials Project, Lawrence Berkeley National Lab, 2016, https://doi.org/10.17188/1189751.

[S3] A. R. H. F. Ettema, R. A. de Groot, C. Haas, and T. S. Turner, Phys. Rev. B **46**, 7363 (1992).

[S4] Asfandiyar, B. Cai, L.-D. Zhao, and J.-F. Li, J. Materiomics **6**, 77 (2020).

[S5] U. Aseginolaza, R. Bianco, L. Monacelli, L. Paulatto, M. Calandra, F. Mauri, A. Bergara, and I. Errea, Phys. Rev. B **100**, 214307 (2019).

[S6] Q. Tan, L.-D. Zhao, J.-F. Li, C.-F. Wu, T.-R. Wei, Z.-B. Xing, and M. G. Kanatzidis, J. Mater. Chem. A **2**, 17302 (2014).

[S7] Narducci, D.; Bermel, P.; Lorenzi, B.; Wang, N.; Yazawa, K. *Hybrid and Fully Thermoelectric Solar Harvesting*, 1st ed. 2018.; Springer Series in Materials Science; Springer International Publishing : Imprint: Springer: Cham, 2018. https://doi.org/10.1007/978-3-319-76427-6.
